# Interlayer gap widened α-phase molybdenum trioxide as high-rate anodes for dual-ion-intercalation energy storage devices

**DOI:** 10.1038/s41467-020-15216-w

**Published:** 2020-03-12

**Authors:** Minghao Yu, Hui Shao, Gang Wang, Fan Yang, Chaolun Liang, Patrick Rozier, Cai-Zhuang Wang, Xihong Lu, Patrice Simon, Xinliang Feng

**Affiliations:** 1 Center for Advancing Electronics Dresden (cfaed) & Department of Chemistry and Food Chemistry, Technische Universität Dresden, 01062 Dresden, Germany; 2 CIRIMAT, Université de Toulouse, CNRS, Toulouse, France; 3 Réseau sur le Stockage Electrochimique de l’Energie (RS2E), CNRS, 3459 Amiens, France; 4 MOE of the Key Laboratory of Bioinorganic and Synthetic Chemistry, The Key Lab of Low-carbon Chem & Energy Conservation of Guangdong Province, KLGHEI of Environment and Energy Chemistry, School of Chemistry, Sun Yat-sen University, 510275 Guangzhou, China; 5 Instrumental Analysis and Research Centre, Sun Yat-sen University, 510275 Guangzhou, China; 6 Ames Laboratory-U. S. Department of Energy, and Department of Physics and Astronomy, Iowa State University, Ames, IA 50011 USA

**Keywords:** Batteries, Materials science, Batteries, Batteries

## Abstract

Employing high-rate ion-intercalation electrodes represents a feasible way to mitigate the inherent trade-off between energy density and power density for electrochemical energy storage devices, but efficient approaches to boost the charge-storage kinetics of electrodes are still needed. Here, we demonstrate a water-incorporation strategy to expand the interlayer gap of α-MoO_3_, in which water molecules take the place of lattice oxygen of α-MoO_3_. Accordingly, the modified α-MoO_3_ electrode exhibits theoretical-value-close specific capacity (963 C g^−1^ at 0.1 mV s^−1^), greatly improved rate capability (from 4.4% to 40.2% at 100 mV s^−1^) and boosted cycling stability (from 21 to 71% over 600 cycles). A fast-kinetics dual-ion-intercalation energy storage device is further assembled by combining the modified α-MoO_3_ anode with an anion-intercalation graphite cathode, operating well over a wide discharge rate range. Our study sheds light on a promising design strategy of layered materials for high-kinetics charge storage.

## Abstract (web summary)

The power/energy trade-off is a common feature seen in a Ragone plot for an electrochemical storage device. Here the authors approach this issue by showing water-incorporated α-MoO_3_ anodes with expanded interlayer gaps, which allow for the assembling of dual-ion energy storage devices.

## Introduction

Triggered by a huge energy demand from various industries ranging from individual electronics to grid storage, electrochemical energy storage devices represent an active field for both research development and practical applications^1–4^. Typically, supercapacitors and batteries differ in electrochemical mechanisms, hence featuring almost opposite energy and power characteristics^5–8^. However, the demand for power and energy supply is equally imperative in actual use and is keen to expand in the future. Thus it is highly desirable to design new electrochemical energy storage technologies to mitigate the trade-off between power density and energy density^9^. Recently, the dual-ion energy storage (DIES) concept has attracted increasing attention as it holds different charge storage chemistry with the conventional “rocking-chair” mechanism^10–13^. In DIES devices, the electrolyte provides both cations and anions to be involved in charge storage. Graphitic carbon provides an ideal host for anion accommodation. The high anion-intercalating potential (>4.0 V vs. Li/Li^+^) and ultrafast rate capability (78% capacity retention, up to 100 C rate) enable graphitic carbon a promising cathode for constructing energy storage devices with both high energy and power densities^14^. Previous studies have proposed several new DIES devices by assembling graphite cathode with conventional battery-type anodes (such as Li metal^14^, graphite^15^, Al metal^16^, Sn metal^17^, WS_2_^18^, etc.). However, the power performance of DIES devices is still limited, and there is great interest in designing high-rate pseudocapacitive anode to be combined with graphite cathode to assemble high-energy, high-power DIES device.

Orthorhombic MoO_3_ (α-MoO_3_) is composed of edge-sharing [MoO_6_] octahedra bilayers stacked along [010] by van der Waals (vdW) interaction. It has been considered as one of the most promising cation-intercalation materials due to its eco-friendliness and also high Mo^6+^/Mo^4+^ redox activity^19,20^. The Li^+^-intercalation reaction in α-MoO_3_ involves both Mo^6+^/Mo^5+^ and Mo^5+^/Mo^4+^ redox couples, which makes α-MoO_3_ a promising electrode for Li-ion storage with a theoretical capacity of 1005 C g^−1^ ^21^. Also, the electrochemical potential for Li-ion intercalation (around 2–3 V vs. Li/Li^+^) is far below the anion-intercalation potential of graphitic carbon (>4.0 V vs. Li/Li^+^), which suggests the promise for assembling DIES devices composed of MoO_3_ anode and graphite cathode. However, the widespread application of α-MoO_3_ is severely restricted by its modest electrochemical reaction kinetics due to the low charge/ion transport efficiency, leading to poor rate capability of α-MoO_3_ electrodes^19,22^. In addition, the unfavorable phase transition of α-MoO_3_ electrode around 2.8 V vs. Li/Li^+^ during the initial lithiation process results in the distortion of layers and a rapid capacity decay during the cycling test^23,24^. To tackle these obstacles, previous studies employed approaches like constructing nanostructures^25^ and creating oxygen vacancy^26^ to obtain favorable α-MoO_3_ electrode with pseudocapacitive charge storage behavior and acceptable cycling life. Nevertheless, those reported α-MoO_3_ electrodes presented unsatisfied areal capacity performance due to low electrode weight loading (e.g., 39 mC cm^−2^ at 1 mV s^−1^ with mass loading of 40 μg cm^−2^)^26^. Such low areal performance limits the practical interest of these electrodes in energy storage devices, since it would result in a drastic decrease in performance at the cell level^27^.

In this study, we widen the interlayer gaps of α-MoO_3_ by incorporating H_2_O molecules between vdW interlayers (α-MoO_3_ with expanded interlayer gaps is denoted as e-MoO_3_). The incorporated H_2_O molecules take the place of lattice O of α-MoO_3_ and expand the ionic channel dimension, which is evidenced by the obviously increased *b*-lattice parameter of e-MoO_3_ (15.02 Å) compared to α-MoO_3_ (13.85 Å). e-MoO_3_ electrode prepared from this approach exhibits a high specific capacity (963 C g^−1^ or 578 mC cm^−2^ at 0.1 mV s^−1^), remarkably improved electrochemical reaction kinetics with boosted rate performance (e-MoO_3_ vs. α-MoO_3_: 40.2% vs. 4.4% capacity retention at 100 mV s^−1^) as well as cycling life (e-MoO_3_ vs. α-MoO_3_: 71% vs. 21% capacity retention over 600 cycles) during Li^+^ storage. Finally, we assemble a DIES device combining a Li^+^-intercalation e-MoO_3_ anode together with a PF_6_^−^-intercalation graphite cathode in 2 M LiPF_6_ electrolyte. Taking advantage of the fast-kinetics intercalation/de-intercalation processes at both anode and cathode, the as-assembled DIES device operates within a 1.0–3.5 V voltage window over a wide discharge rate range (discharge time from 25 s to 3.5 h), and shows high energy density (up to 44 Wh L^−1^ based on the whole device) with decent power capability (600 W L^−1^).

## Results

### Expanding the interlayer gaps of α-MoO_3_

**Fig. 1:**
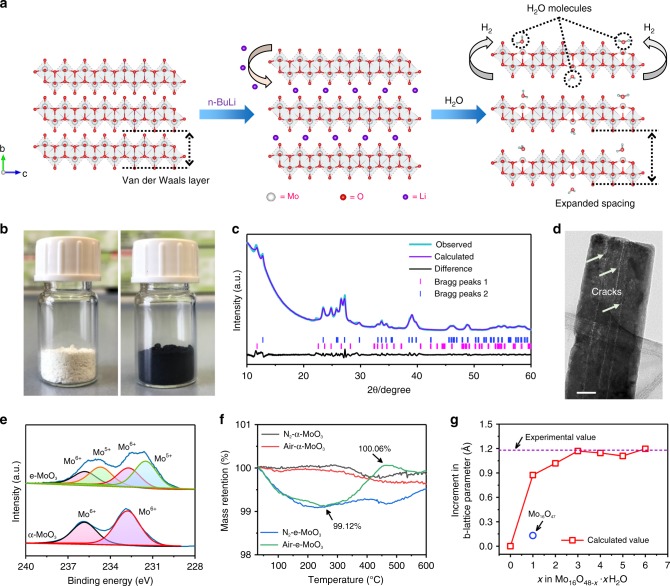
Structure and composition analysis of α-MoO_3_ and e-MoO_3_. **a** Schematic representation of expansion process from pristine α-MoO_3_ (left), reaction with *n*-butyllithium (middle), to e-MoO_3_ with H_2_O molecules located at the oxygen-defect sites (right). White: Mo; Red: O; Purple: Li; Blue: H. **b** Digital photos of α-MoO_3_ (left) and e-MoO_3_ (right). **c** Rietveld refinement of XRD pattern and **d** high-resolution TEM image for e-MoO_3_.Scale bar in **d** 50 nm. **e** Mo 3d XPS profiles of α-MoO_3_ and e-MoO_3_. **f** TGA curves of α-MoO_3_ and e-MoO_3_ measured in nitrogen and air atmosphere. **g** Calculated increment in *b*-lattice parameter induced by H_2_O intercalation. The value is also compared with the calculated value of oxygen-defective MoO_3−*x*_ (Mo_16_O_47_) and experimental value.

The procedure for expanding interlayer gaps of pristine α-MoO_3_ is described in Fig. 1a. α-MoO_3_ nanowires were first synthesized through a hydrothermal method and further immersed for 12 h in a solution of *n*-butyllithium dissolved in hexane. Fast color change from white to dark blue was observed (Fig. 1b), suggesting the creation of reduced Mo species due to Li^+^ intercalation^19,26^. Afterwards, the sample was transferred into water, resulting in the oxidation of the sample by Li^+^ removal and simultaneous reduction of water into gaseous hydrogen. Thus the combination of reduction using *n*-butyllithium and oxidation using H_2_O leads to a complex process enlisting also a replacement of lattice oxygen by some H_2_O molecules, which enforces the expansion of the interlayer gaps.

The comparison of X-ray diffraction (XRD) patterns confirms the expansion of the interlayer space. The diffraction data of pristine α-MoO_3_ (Supplementary Fig. 1 and Supplementary Note 1) are indexed to JCPDS card no. 05-0508. It crystallizes in the orthorhombic system (SG: Pbnm) with the refined lattice parameters of *a* = 3.96 (6) Å, *b* = 13.85 (1) Å, *c* = 3.70 (5) Å. e-MoO_3_ maintains similar peaks of α-MoO_3_ with relatively broad peak width and, more importantly, shows (0*k*0) peaks shifted toward lower angles (Fig. 1c). These peaks refer to an enlarged *b* value of 15.02 (3) Å, while *a* and *c* parameters stay almost unchanged (*a* = 4.08 (3) Å, *c* = 3.75 (2) Å). This result indicates an impressive expansion of the interlayer distance by 0.6 Å, largely higher than the 0.1 Å expansion reported for oxygen deficient α-MoO_3_ compound^26^. Thus the wide interlayer distance in e-MoO_3_ reported here is far above the interlayer distance solely induced by the oxygen vacancies. High-resolution transmission electron microscopic (TEM) and scanning electron microscopic (SEM) images (Fig. 1d and Supplementary Fig. 2) confirm that e-MoO_3_ retains the nanowire structure but with a rougher surface with numerous cracks, in agreement with mechanical strains induced by the large expansion of interlayer space.

To elucidate the composition evolution along with interlayer gap expansion, Fourier transform infrared (FTIR) and Raman measurements have been conducted for α-MoO_3_ and e-MoO_3_ (Supplementary Fig. 3 and Supplementary Note 2). Compared with the FTIR and Raman peaks of MoO_3_, the remarkable change observed for e-MoO_3_ indicates the obvious crystal re-arrangement of the [MoO_6_] octahedra bilayers in e-MoO_3_. Electron paramagnetic resonance (EPR) spectra of both α-MoO_3_ and e-MoO_3_ were measured at a low temperature of 90 K (Supplementary Fig. 4). In contrast to α-MoO_3_, e-MoO_3_ displays an apparent signal with a *g* value of 1.93 and a line width of 49 Gs, which can be assigned to the presence of Mo^5+^ donor levels close to the conduction band^28,29^. Consistent result is identified in Mo 3d X-ray photoelectron spectroscopy (XPS) data of e-MoO_3_ with newly appeared Mo^5+^ 3d 3/2 and 3d 5/2 peaks (234.7 and 231.5 eV, respectively, Fig. 1e)^19,26^. The atomic ratio between Mo^5+^ and Mo^6+^ reaches approximately 3:2. Comparison of O 1s XPS spectra (Supplementary Fig. 5) shows the presence of an extra peak at 532.4 eV for e-MoO_3_ in addition to the peak located at 530.7 eV observed in α-MoO_3_ which is characteristic of lattice oxygen^30^. This confirms the presence of incorporated H_2_O in e-MoO_3_ sample, and the atomic percentage of O in the incorporated H_2_O accounts for about 10% of total O atoms. No Li 1s signal is detected for e-MoO_3_, ruling out the possible pre-lithiation in e-MoO_3_ (Supplementary Fig. 6). This is consistent with the XRD patterns of e-MoO_3_ after annealing in either N_2_ or air, where no Li-containing compound is observed (Supplementary Fig. 7 and Supplementary Note 3). Thus, based on XPS analyses, the surface composition of e-MoO_3_ can be roughly estimated as MoO_2.7_·0.3 H_2_O. Thermogravimetry analysis (TGA, Fig. 1f) of e-MoO_3_ in the air was conducted to estimate the amount of incorporated H_2_O in the whole sample. It presents the loss of intercalated water occurring at ~250 °C (mass retention of 99.12%) and the re-oxidation of oxygen-deficient MoO_3_ in the temperature range of 250–460 °C (mass retention of 100.06%), which is indicative of the formula MoO_2.92_·0.07 H_2_O for e-MoO_3_. From both XPS and TGA analysis, we notice that the molar amount of oxygen defects is approximately equal to that of the incorporated H_2_O molecules. Thus we consider that the incorporated H_2_O molecules take the place of lattice O of e-MoO_3_, functioning as buffered spacer to stabilize the widened interlayer gaps between the layers. This assumption is further supported by the density functional theory (DFT) simulation, which is performed employing the bulk supercells of Mo_16_O_48−*x*_·*x*H_2_O (*x* = 1–6, which represents the number of the incorporated H_2_O molecules) shown in Supplementary Figs. 8 and 9. Notably, an increment of 0.9 Å in the *b*-lattice parameter is revealed even when one lattice O is replaced by one H_2_O molecule (Fig. 1g). By contrast, for the sample with only oxygen defects (Mo_16_O_47_), the increment can only reach 0.13 Å, which further confirms the dominant role of the incorporated H_2_O in expanding the interlayer gaps. When *x* is between 3 and 6 (corresponding to MoO_2.81_·0.19 H_2_O to MoO_2.63_·0.37 H_2_O), the calculated increment in the *b*-lattice parameter agrees well with our experimental XRD results. In addition, the simulated e-MoO_3_ structure with most stable configuration also reveals that the incorporated H_2_O induces the distortion of the [MoO_6_] octahedra (Supplementary Fig. 10), leading to substantial changes in O–O distances (both intra- and inter-layer ones, Supplementary Fig. 11 and Supplementary Note 4).

The increment in band density near the Fermi level for e-MoO_3_ compared with α-MoO_3_ is evidenced by the ultraviolet photoelectron spectroscopy analysis (Supplementary Figs. 12 and 13 and Supplementary Note 5) and O *K*-edge X-ray absorption near-edge structure spectra (Supplementary Fig. 14 and Supplementary Note 6). e-MoO_3_ sample exhibits a remarkable conductivity of 0.95 S m^−1^ at 300 K as determined by the van der Pauw 4-probe method (Supplementary Fig. 15), whereas the conductivity of pristine α-MoO_3_ was too low to be detected. The enhanced conductivity can be explained by the narrowed band gap and the increment in the carrier concentration (Supplementary Fig. 16 and Supplementary Note 7), which can be attributed to the electron hopping mechanism boosted by Mo^5+^ ^31,32^.

### Enhanced Li^+^ storage kinetics of e-MoO_3_

**Fig. 2:**
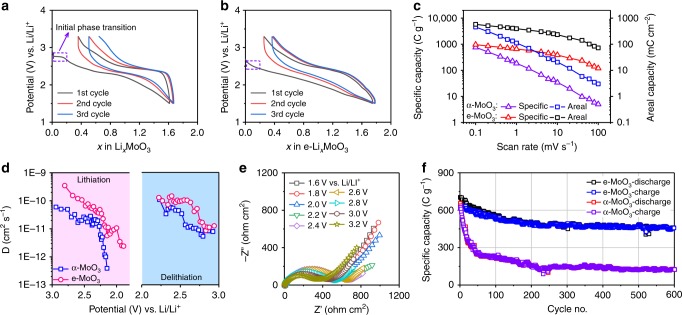
Li^+^ storage behavior of α-MoO_3_ and e-MoO_3_ electrodes. Initial three galvanostatic charge/discharge profiles of **a** α-MoO_3_ and **b** e-MoO_3_ electrodes. **c** Specific and areal capacity as a function of scan rate. **d** Li^+^ diffusion coefficient calculated from GITT curves. **e** Nyquist plots of e-MoO_3_ at different potentials. **f** Cycling performance of α-MoO_3_ and e-MoO_3_ at 100 mA g^−1^.

e-MoO_3_ sample with large opened interlayer space has been used as electrode material for Li-ion intercalation reaction. Figure 2a, b compares the initial three galvanostatic charge/discharge cycles at 50 mA g^−1^ in 2 M LiPF_6_ dissolved in ethyl methyl carbonate electrolyte. The first lithiation process of α-MoO_3_ starts with a short constant potential plateau at about 2.75 V vs. Li/Li^+^, which is consistent with the presence of a reduction peak in the first cathodic cyclic voltammetry (CV) cycle (Supplementary Fig. 17). This process is related to an irreversible phase transition and will be later discussed in more detail in the in operando XRD section. By contrast, such a feature is not observed in the first lithiation reaction of e-MoO_3_, identifying the role of the widened interlayer gaps in inhibiting the phase transition. Also, the Coulombic efficiency of e-MoO_3_ in the first cycle (85%) is found to be higher than that of α-MoO_3_ (78%). Such an improvement is also observed during the next cycles (Fig. 2a, b).

CV curves from a scan rate range of 0.1–100 mV s^−1^ were collected for both α-MoO_3_ and e-MoO_3_ electrodes with similar weight loadings (Supplementary Fig. 18). As revealed in Fig. 2c, e-MoO_3_ depicts both higher capacity and better rate capability than α-MoO_3_. At an extremely slow scan rate of 0.1 mV s^−1^, the capacity of e-MoO_3_ electrode is close to the theoretical capacity (1005 C g^−1^, 279 mAh g^−1^), while α-MoO_3_ electrode only shows a capacity of 756 C g^−1^ (210 mAh g^−1^). Importantly, our e-MoO_3_ electrode delivers an outstanding areal capacity of 578 mC cm^−2^, drastically outperforming the reported state-of-the-art α-MoO_3_-based pseudocapacitive electrodes^19,22,25,26,33^. When the scan rate increases to 10 and 100 mV s^−1^, e-MoO_3_ can maintain high capacities of 233 and 72 mC cm^−2^, respectively, indicating high power capability. As for α-MoO_3_ electrode, the capacity at 10 and 100 mV s^−1^ can only reach 20 and 3 mC cm^−2^, respectively. Moreover, galvanostatic charge/discharge profiles at different rates also verify the superior rate capability of e-MoO_3_ electrode (Supplementary Fig. 19).

The significant role of the incorporated H_2_O molecules in accelerating the diffusion of Li^+^ within e-MoO_3_ lattice was further studied. Figure 2d compares the Li^+^ diffusion coefficient (*D*) of α-MoO_3_ and e-MoO_3_ obtained by employing a galvanostatic intermittent titration technique (GITT, see Supplementary Figs. 20 and 21). The *D*_Li+_ values in e-MoO_3_ electrode (2.4 × 10^−12^–3.5 × 10^−10^ cm^2^ s^−1^) are substantially higher than those in α-MoO_3_ electrode (3.9 × 10^−13^–6.0 × 10^−11^ cm^2^ s^−1^). The fastened Li^+^ diffusion can be assigned to both the expanded ionic diffusion channels and the shielding effect of incorporated H_2_O that screens the electrostatic interaction between Li^+^ and MoO_3_ lattice^34,35^. Meanwhile, we measured the electrochemical impedance spectroscopy for both α-MoO_3_ (Supplementary Fig. 22) and e-MoO_3_ (Fig. 2e) at various potentials from the cathodic process. Both electrodes depict stable ohmic resistance (*R*_s_) and V-shape variation for charge transfer resistance (*R*_ct_) along with the change of potential (Supplementary Fig. 23). It can be observed that *R*_*s*_, *R*_ct_, and the overall resistance of e-MoO_3_ are all lower than those of α-MoO_3_, further justifying the improved charge and ion transfer efficiency for e-MoO_3_.

In addition, e-MoO_3_ electrode exhibits a significant improvement in cycling stability compared to α-MoO_3_ (Fig. 2f). When cycled at 100 mA g^−1^ for 600 cycles, e-MoO_3_ still maintains a high capacity of 455 C g^−1^, delivering excellent capacity retention of 71%. In contrast, α-MoO_3_ electrode shows rapid capacity decay, from 586 C g^−1^ in the first cycle to 175 C g^−1^ after only 50 cycles.

**Fig. 3:**
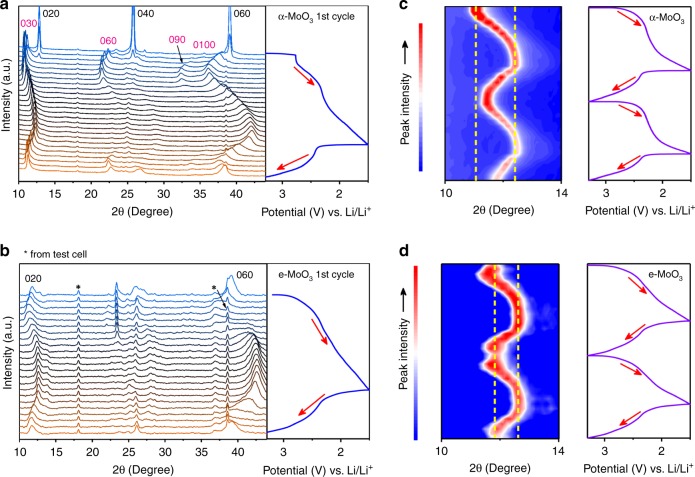
In operando XRD study. In operando XRD measurements of **a**, **c** α-MoO_3_ and **b**, **d** e-MoO_3_ electrodes during **a**, **b** the first galvanostatic lithiation/de-lithiation cycle and **c**, **d** the second and third cycles.

To gain insight into the performance superiority of e-MoO_3_ to pristine α-MoO_3_, we further carried out in operando XRD characterizations of electrodes during the first three lithiation/de-lithiation cycles. During the initial lithiation (Fig. 3a), α-MoO_3_ electrode experienced an irreversible phase transition at 2.75 V vs. Li/Li^+^, which was evidenced by the rapid disappearance of the (0*k*_1_0) peaks of the pristine α-MoO_3_ and the appearance of a set of broad (0*k*_2_0) peaks belonging to the lithiated MoO_3_ (Li_*x*_MoO_3_). This irreversible phase transition results in a sudden distortion of the layers in α-MoO_3_, which accounts for the fast structural collapse and sluggish kinetics of α-MoO_3_^36^. Although limited information can be gained for e-MoO_3_ from XRD peaks within 15–35° due to the overlap of multiple peaks, one can clearly notice that the primitive (020) and (060) peaks of e-MoO_3_ were well maintained during the whole lithiation/de-lithiation process (Fig. 3b). This result suggests a stable structural phase resulting from the expanded interlayer gaps in e-MoO_3_. We further compare the shift of the (0*k*0) peak of α-MoO_3_ and e-MoO_3_ located between 10° and 14° during the second and third lithiation/de-lithiation cycles (Fig. 3c, d), which is directly associated with the volume change of the lattice in *b* direction. Benefiting from the lessened electrostatic interaction between Li^+^ and MoO_3_ lattice, the peak shift of e-MoO_3_ (0.8°) during the second cycle is apparently smaller than that of α-MoO_3_ (1.3°). Besides, almost no difference is observed between the peaks of e-MoO_3_ at the beginning of the second cycle and at the end of the third cycle, while α-MoO_3_ displays a peak shift of 0.6°. Thus we conclude that the incorporated H_2_O molecules efficiently alleviate the volume change of e-MoO_3_ and prevent the structure collapse during Li^+^ insertion/extraction, which also explains the enhanced cycling performance of e-MoO_3_ with respect to α-MoO_3._ Moreover, aside from α-MoO_3_, we also demonstrated the feasibility of our H_2_O-incorporation strategy in improving the charge storage ability of birnessite δ-MnO_2_ for Zn-ion aqueous batteries (Supplementary Figs. 24 and 25 and Supplementary Note 8).

### Full DIES device

**Fig. 4:**
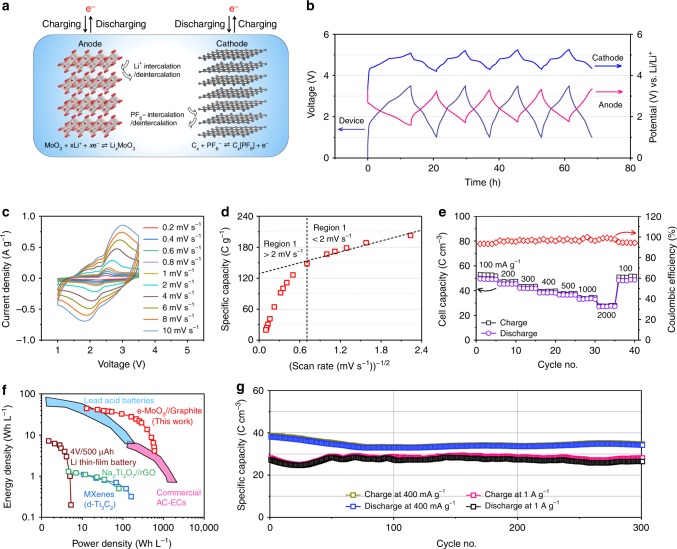
e-MoO_3_//graphite DIES device. **a** Design of the dual-ion-intercalation MoO_3_//graphite cell. On the anode side, predominantly Li^+^ is intercalated and de-intercalated between [MoO_6_] layers of MoO_3_ during charge/discharge. On the cathode side, graphite is used as a host for hexafluorophosphate (PF_6_^−^) anion intercalation/de-intercalation. **b** The initial four galvanostatic charge/discharge cycles at 100 mA g^−1^ and the corresponding potential variation of the cathode and anode. **c** CV curves of the device at different scan rates. **d** Change of the gravimetric capacity based on the total active mass as a function of *v*^−1/2^. **e** Rate performance of the e-MoO_3_//graphite device based on the volume of the whole device. **f** Ragone plots of e-MoO_3_//graphite device in comparison with state-of-the-art electrochemical energy storage devices; the performance is normalized to the total cell volume, which includes the active materials, current collectors and separators. **g** Cycling performance of the e-MoO_3_//graphite device at 400 mA g^−1^ and 1 A g^−1^.

Full DIES device was further assembled by e-MoO_3_ anode, graphite cathode and 2 M LiPF_6_ in ethyl methyl carbonate electrolyte to demonstrate the practical application of our e-MoO_3_ electrode in constructing full device. As anion-intercalation graphite cathode owns a high anion-intercalation potential (average discharge potential: 4.6 V), a large specific capacity (396 C g^−1^ at 50 mA g^−1^), and importantly high charge-storage kinetics (almost no capacity decay from 50 to 500 mA g^−1^, Supplementary Fig. 26 and Supplementary Note 9), the designed e-MoO_3_//graphite device holds the opportunity to mitigate the inherent trade-off between energy density and power density for energy storage devices. According to the specific capacity of e-MoO_3_ anode and graphite cathode at a low rate, a cathode/anode mass ratio of 2.5 was used to assemble the dual-ion-intercalation device (Fig. 4a), and a theoretical capacity of 275 C g^−1^ is expected for the cell based on the total mass of active materials. From the experiments, a reversible capacity of 200 C g^−1^ (72.7% of theoretical capacity) could be achieved at 100 mA g^−1^ (based on the mass of e-MoO_3_) within a 1.0–3.5 V voltage range.

Figure 4b shows the first four galvanostatic charge/discharge cycles using a Li metal reference electrode to record the anode and cathode potentials during the cell cycling. After the first two cycles, the potential windows for anode and cathode stabilize as 1.8–3.3 and 4.3–5.3 V vs. Li/Li^+^, respectively (Fig. 4b). At the end of charge and discharge, the cathode and anode are close to its upper potential limit (3.3 V vs. Li/Li^+^ for e-MoO_3_, 5.3 V vs. Li/Li^+^ for graphite), respectively. The unexploited capacity (capacity at a potential <1.8 V for e-MoO_3_ and <4.3 V for graphite) explains the lower capacity of the device compared with the theoretical values. Noteworthy is that the Coulombic efficiency of e-MoO_3_ anode (85%) at the first cycle is close to that of graphite cathode (75%, Supplementary Fig. 27), so that the two electrodes in the full cell provide suitable buffers for each other during the initial polarization. Moreover, the anode potential is far above the Li plating potential, which can effectively prevent the safety issue associated with metal dendrite growth.

CV curves recorded from 0.2 to 100 mV s^−1^ (discharge time from 3.5 h to 25 s) present multi-pairs of redox peaks, corresponding to different steps of anion and cation intercalation/de-intercalation (Fig. 4c and Supplementary Fig. 28). To establish the rate-limiting step of the charge storage process, Fig. 4d plots the specific capacity based on the total active mass as a function of *v*^−1/2^ ^37^. At a low scan rate of 0.2 mV s^−1^, the capacity is up to 222 C g^−1^. Along with the increasing scan rate, there are two distinct regions. At scan rates <2 mV s^−1^, the charge storage is almost not limited by the solid-state ion diffusion, thus the capacity is mostly independent on the scan rate. The extrapolated *y*-intercept capacity, that is, the charge storage from capacitive processes at the infinite scan rate, reaches around 130 C g^−1^. The high kinetics of the device is also reflected by the slope of the plots of log (*i*) vs. log (*v*) for the main redox peaks (Supplementary Fig. 29 and Supplementary Note 10).

We further studied the rate performance of the DIES device from 100 mA g^−1^ to 2 A g^−1^ (Fig. 4e). The capacity based on the total cell stack volume (illustrated in Supplementary Fig. 30 and Supplementary Note 11) reaches 49 C cm^−3^ (200 C g^−1^, Supplementary Fig. 31) at 100 mA g^−1^, and 55% capacity was maintained when the current density increased to 2 A g^−1^ (a discharge time of 3.2 min). The Ragone plot shown in Fig. 4f compares the volumetric performance of the cell with the state of the art of electrochemical energy storage devices (Supplementary Fig. 32 shows the performance normalized to the active material weight). The present e-MoO_3_//graphite device could deliver an energy density up to 44 Wh L^−1^, which outperforms those of Li thin-film micro-battery^38^, Na-ion capacitors^39^, activated carbon-^40^ and MXene-based^41^ supercapacitors, and is comparable to those of lead-acid batteries^42^. Meanwhile, a decent maximum power density of 600 W L^−1^ was achieved, close to supercapacitor-level power densities.

Finally, cycling stability was evaluated by galvanostatic charge/discharge tests (Fig. 4g). Impressive cycling stability with 90% and 97% capacity retention was observed over 300 cycles at 400 and 1000 mA g^−1^. Note that both the volumetric and gravimetric capacities of graphite are substantially lower than those of e-MoO_3_, which consequently limit the energy storage capability of the whole DIES device. Li-ion full cell was also assembled by e-MoO_3_ anode and commercial LiCoO_2_ cathode (Supplementary Fig. 33 and Supplementary Note 12). Although the maximum energy density of e-MoO_3_//LiCoO_2_ device (146 Wh kg^−1^) is slightly higher than that of e-MoO_3_//graphite device (133 Wh kg^−1^), the power density of e-MoO_3_//LiCoO_2_ is far below that of e-MoO_3_//graphite device. As a result, developing a new cation-intercalation cathode with comparable specific capacity with e-MoO_3_ anode would be beneficial for further boosting the energy and power densities of full devices.

## Discussion

In summary, we demonstrated an efficient H_2_O-incorporation strategy for expanding the interlayer gaps of α-MoO_3_, which triggers a robust Li^+^ storage ability with significantly improved rate capability and prolonged cycling stability. There are three key advantages of our strategy. First, the incorporated H_2_O molecules function as effective spacers to stabilize the expanded interlayer gaps in e-MoO_3_, which provides efficient Li^+^ diffusion channels and avoid the irreversible phase transition of e-MoO_3_ during the initial lithiation process. Second, the H_2_O molecules can shield electrostatic interaction between Li^+^ and e-MoO_3_ lattice, thus alleviating the volume change of e-MoO_3_ during repeated Li intercalation/de-intercalation and improving the cycling stability of e-MoO_3_. Third, since the incorporated H_2_O molecules take the place of lattice O and the molecular mass of H_2_O is very close to O, the improved rate capability and cycling stability of e-MoO_3_ come without the sacrifice of specific capacity. Moreover, a DIES device was assembled by coupling cation-intercalation e-MoO_3_ and anion-intercalation graphite. Such a device links the intercalation-type charge storage with fast kinetics, which thus can bring together high power density (up to 600 W L^−1^) and energy density (up to 44 Wh L^−1^). This study offers insightful understanding of the interlayer gap-engineering strategy to improve the ion-intercalation chemistry of layered electrode materials, which opens opportunities for the next-generation energy storage technologies.

## Methods

### Materials

All chemicals were of analytical grade and used directly without any purification. Ammonium molybdate tetrahydrate ((NH_4_)_6_Mo_7_O_24_·4H_2_O), lithium cobalt oxide (LiCoO_2_), concentrated HNO_3_ (70 wt%), n-butyl lithium in hexane, hexane, Li foil, graphite flakes (average size 20 µm), sodium alginate, Super P carbon black, and 2 M LiPF_6_ in ethyl methyl carbonate were purchased from Sigma-Aldrich.

### Material preparation

α-MoO_3_ nanowires were prepared through a hydrothermal method. In all, 6.5 g of (NH_4_)_6_Mo_7_O_24_·4H_2_O was first dissolved into 180 mL of distilled water. Then 30 mL concentrated HNO_3_ (70 wt%) was added into the solution, followed by vigorous stirring. The obtained solution was then transferred into a 250-mL Teflon-lined stainless autoclave and heated in an electric oven at 180 °C for 12 h. After naturally cooled down to room temperature, the obtained powder sample was washed thoroughly with distilled water and dried in a vacuum oven at 80 °C for 12 h.

e-MoO_3_ was obtained by dispersing 500 mg of α-MoO_3_ nanowires in 16 mL of 0.8 M n-butyl lithium in hexane. The mixture was then stirred for 24 h at room temperature under the protection of inert argon atmosphere. The lithiated compound was separated by vacuum filtration and washed several times with hexane. Subsequently, the sample was placed in 100 mL of distilled water and stirred for half an hour. Finally, the sample was centrifuged and dried in a vacuum oven at 80 °C for 12 h.

### Electrochemical measurements

All the half-cell tests were conducted in CR2032 coin-type cells. To prepare the electrodes, active materials (α-MoO_3_, e-MoO_3_, and graphite), binder (sodium alginate), and Super P carbon black were mixed with a weight ratio of 8:1:1. The mixture was dispersed in water and coated on a Cu foil (α-MoO_3_, e-MoO_3_) or an Al foil (graphite), followed by drying at 80 °C for 12 h under vacuum. The loading mass of α-MoO_3_ and e-MoO_3_ is about 0.6 ± 0.1 mg cm^−2^, while the loading mass of graphite is 1.5 ± 0.1 mg cm^−2^. The coin cells were assembled using Li metal foil as a counter electrode, Celgard 2400 as the separator, and 2 M LiPF_6_ dissolved in ethyl methyl carbonate as the electrolyte.

The full-cell tests were performed with a three-electrode stainless steel Swagelok cell. e-MoO_3_ coated on Cu foil and graphite coated on Al foil were used as anode and cathode, respectively, with a mass ratio of 1:2.5. A Li metal foil was inserted as a reference electrode to monitor the potential variation of both anode and cathode. All cell assembly was carried out in an argon-filled glove box, and the concentration of O_2_ and H_2_O were kept <0.1 ppm.

The charge–discharge curves were collected from a Land battery test system (LAND CT2001A) at room temperature. GITT test was performed with a galvanostatic charge/discharge pulse of 50 mA g^−1^ for 10 min, followed by an open circuit step for 3 h. Both CV and electrochemical impedance measurements were conducted on a VMP3 potentiostat (Biologic, USA). The electrochemical impedance measurements were carried out at a 10 mV ac oscillation amplitude over the frequency range of 100 kHz to 0.01 Hz.

### Characterization

The microstructures and compositions of samples were analyzed by field-emission SEM (Carl Zeiss Gemini 500), TEM (FEI Tecnai G^2^ F30), XRD (Bruker D8) using Cu Kα radiation (*λ* = 0.154 nm), and TGA (Netzsch DSC-204 F1). Raman spectra were measured on a NTMDT confocal spectrometer with a 532-nm laser, and the spot size of the laser beam was ~0.5 μm. Infrared spectra were recorded on a FT-IR Spectrometer Tensor II (Bruker) with an ATR unit. EPR measurement was performed in the *X*-band (9.32 GHz) with 1.0 G modulation amplitude and a magnetic field modulation of 100 kHz using a Bruker, A300-10-12 Bruker EPR spectrometer at 90 K.

XPS, near-edge X-ray absorption fine structure (NEXAFS), and ultraviolet photoemission spectroscopy were measured at the Photoemission Endstation (BL10B beamline) of the National Synchrotron Radiation Laboratory (NSRL) in Hefei, China. Briefly, the beamline is connected to a bending magnet and equipped with three gratings that cover photon energies from 100 to 1000 eV with a typical photon flux of 1 × 10^10^ photons/s and a resolution (*E*/*ΔE*) better than 1000 at 244 eV. XPS spectra were collected using monochromatic Mg-Kα X-ray (1254.6 eV) as the excitation source. NEXAFS spectra were recorded in the total electron yield mode using a sample current measurement.

To detect the conductivity, 10 mg sample was first dispersed in ethanol and coated on a cellulose membrane (diameter of 4.5 cm) through vacuum filtration. The membrane loaded with sample was cut into a size of 1 × 1 cm^2^, and then a four-point contact was placed on the surface to define a square. The *I*–*V* curves were measured in van der Pauw geometry under vacuum at varied temperatures (from 100–400 K) using a commercial Lakeshore Hall System.

A two-electrode Swagelok cell was used for in operando XRD tests. Freestanding α-MoO_3_ or e-MoO_3_ film consisting of 90 wt% of active material, 5 wt% of Super P, and 5 wt% of polytetrafluoroethylene was placed onto a beryllium foil acting as the current collector, and a Li foil was used as the counter electrode. XRD test was carried out on a Bruker D8 advance diffractometer in the 10–50° 2*θ* range with a step of 0.0152°, while the electrode was undergoing a galvanostatic charge/discharge test at 50 mA g^−1^.

### Computational details

All calculations were performed using the plane-wave DFT code available in the Vienna Ab initio Simulation Package (VASP) within the generalized gradient approximation, using the Perdew–Burke–Ernzerhof functional for the exchange and correlation potential. Spin polarized calculations were performed to account for orbit coupling. The electronic wave functions were described in the projected augmented wave formalism, and a real-space projection was further used for the total wavefunction analysis. The cutoff energy for plane-wave basis set was set to 400 eV. All atomic positions and lattice vectors were fully optimized using a conjugate gradient algorithm to obtain the unstrained configuration. Atomic relaxation was performed until the change of total energy was <1 × 10^−5^ eV, and all the forces on each atom were <0.01 eV Å^−1^. The orthorhombic cell with space group Pbnm was used for α-MoO_3_. Supercells containing 2 × 1 × 2 conventional cells (16 formula units, Supplementary Figs. 7 and 8) were used to calculate energies and band structure, in which Monkhorst–Pack scheme *k*-point grid was set to 5 × 3 × 5.

## Supplementary information

Supplementary Information

Peer Review File

## Data Availability

The data that support the findings of this study are available from the corresponding authors upon reasonable request.
