# Supplementary material for: Interlayer gap widened α-phase molybdenum trioxide as high-rate anodes for dual-ion-intercalation energy storage devices
Source: Nat Commun. 2020 Mar 12;11:1348. doi: 10.1038/s41467-020-15216-w (PMC7067814; doi:10.1038/s41467-020-15216-w)
Supplement: Supplementary file 1 — Supplementary Information [file 41467_2020_15216_MOESM1_ESM.pdf]

Supplementary Information

Interlayer Gap Widened α-Phase Molybdenum Trioxide as High-Rate Anodes for Dual-Ion-Intercalation Energy Storage Devices

Yu *et al.*

Supplementary Figures

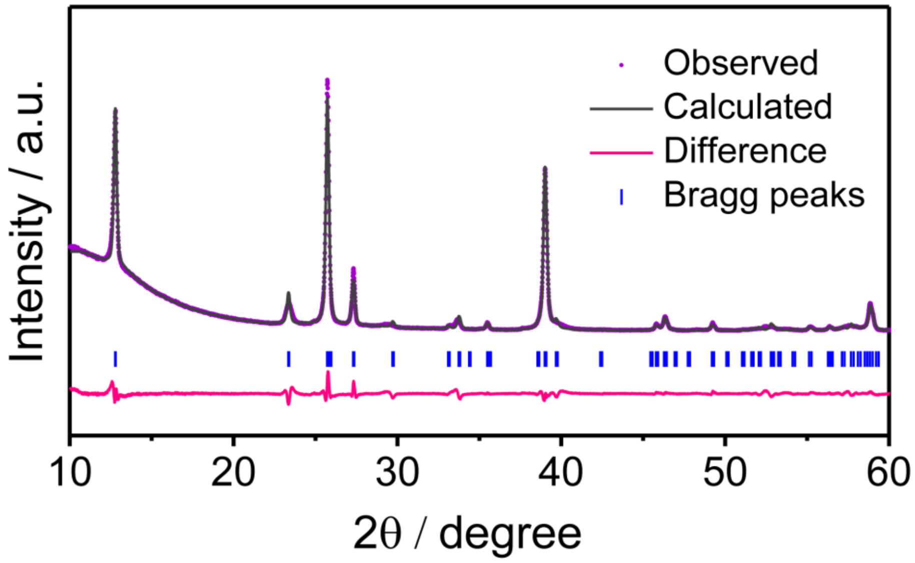

Supplementary Figure 1 Rietveld refinement of XRD pattern for α-MoO_3.

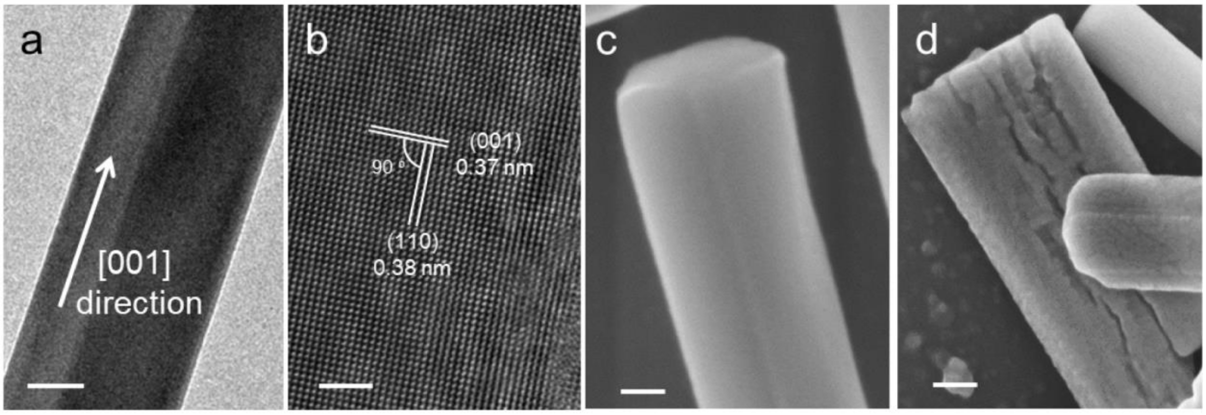

Supplementary Figure 2 TEM and SEM images. (a) Low-resolution and (b) high-resolution TEM images of α-MoO_3 nanowire. Scale bar in (a), 50 nm. Scale bar in (b), 3 nm. SEM images of (c) α-MoO_3 and (d) e-MoO_3. Scale bars in (c, d), 100 nm. In comparison with the smooth surface of α-MoO_3, clear cracks were observed for e-MoO_3.

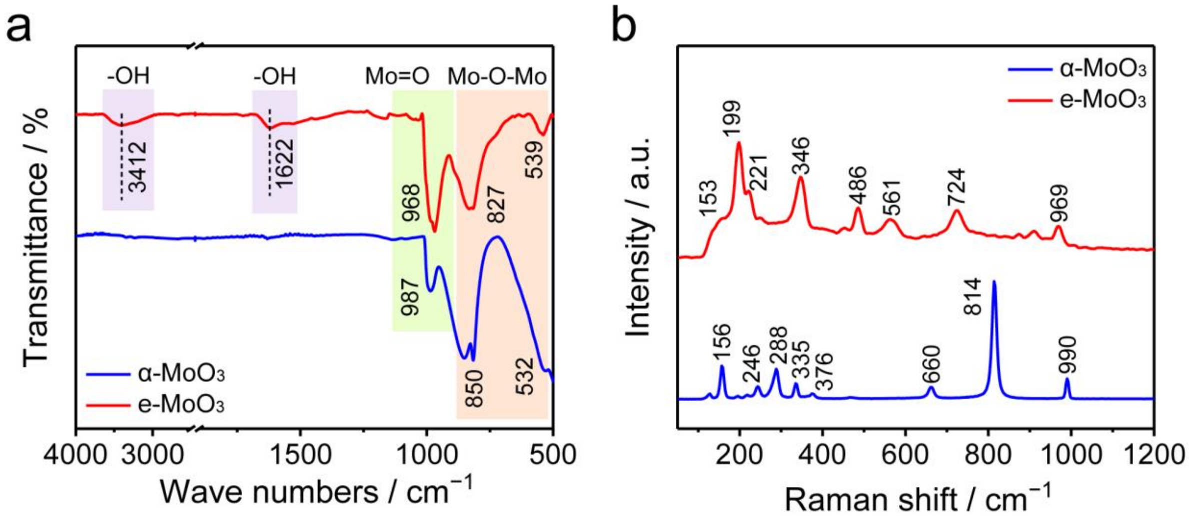

Supplementary Figure 3 Characterizations of α-MoO_3 and e-MoO_3. (a) FTIR and (b) Raman.

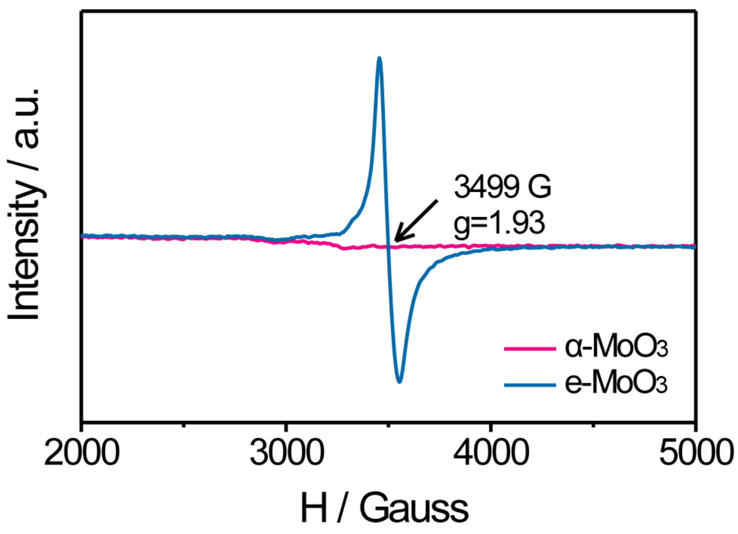

Supplementary Figure 4 EPR spectra of α-MoO_3 and e-MoO_3.

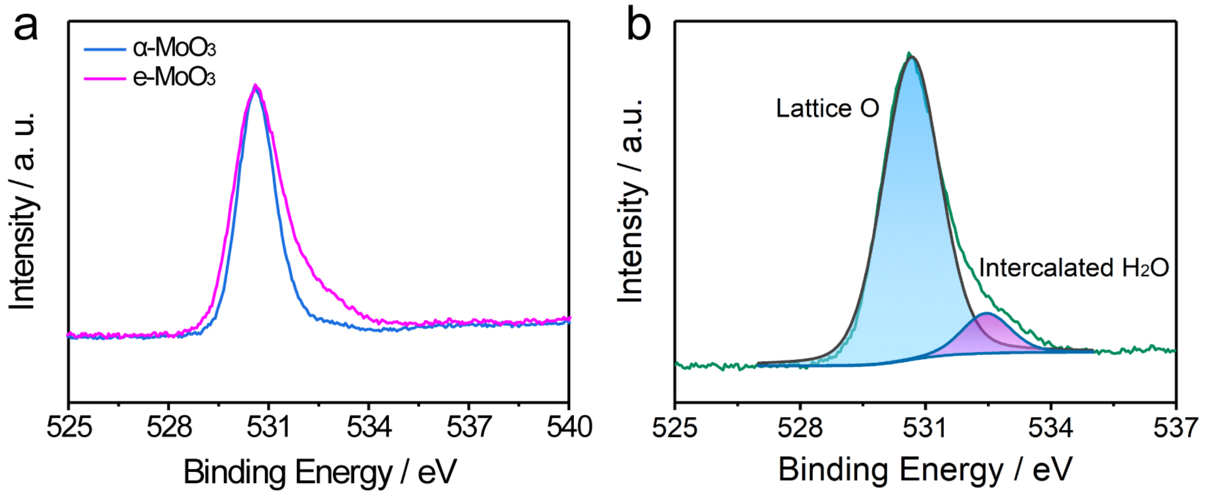

Supplementary Figure 5 XPS spectra. (a) O 1s XPS spectra of α-MoO_3 and e-MoO_3. (b) O 1s XPS spectrum of e-MoO_3, which is de-convoluted into peaks of lattice O (530.7 eV) and intercalated H_2O (531.8 eV).

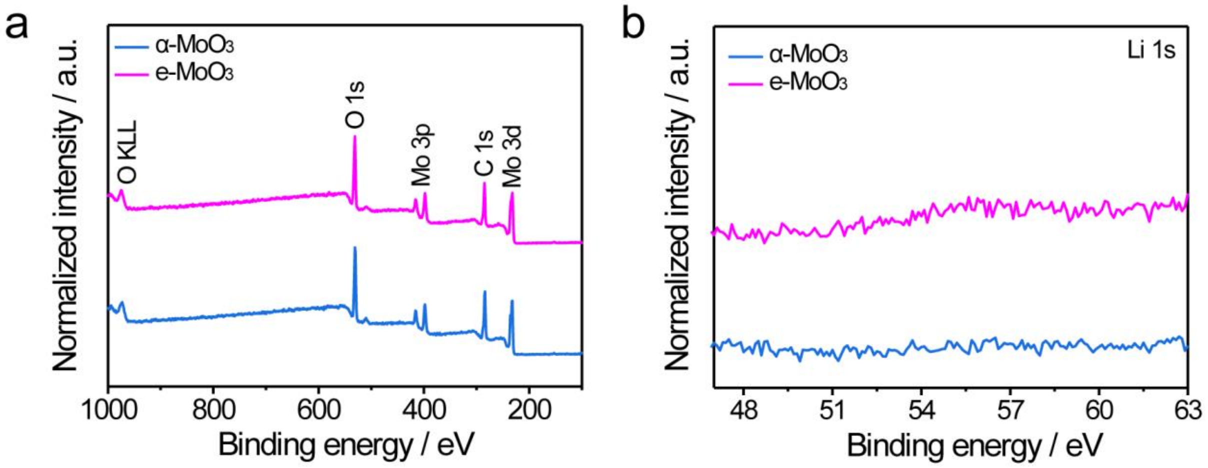

Supplementary Figure 6 XPS spectra. (a) Survey and (b) Li 1s XPS spectra of α-MoO_3 and e-MoO_3.

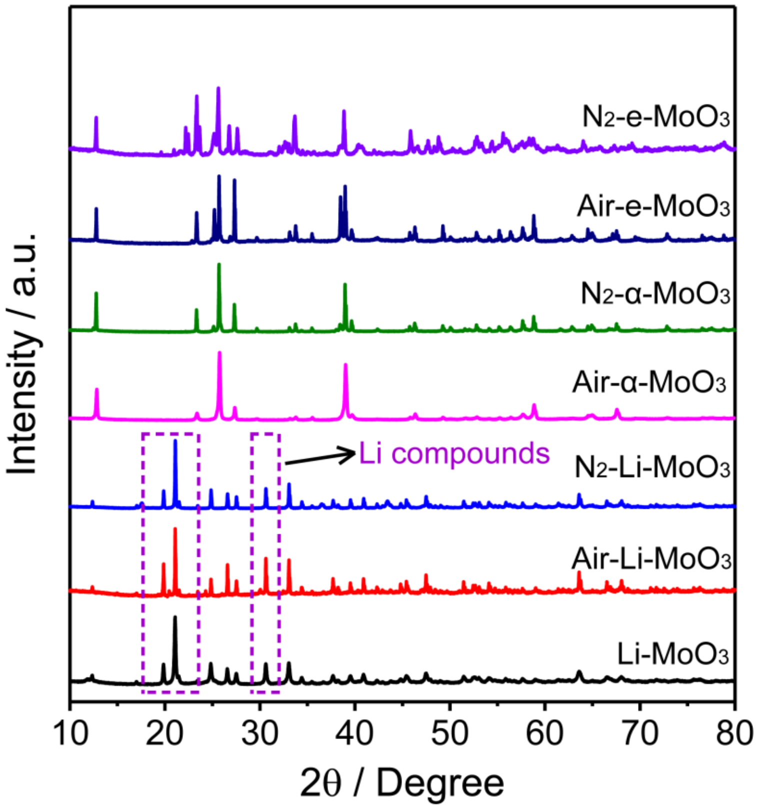

Supplementary Figure 7 XRD patterns of α-MoO_3, lithiated α-MoO_3 and e-MoO_3 samples after thermal treatment at 500 °C in N_2 or air atmosphere. Li-MoO_3 refers to lithiated α-MoO_3, which was obtained by dispersing α-MoO_3 in n-butyl lithium solution in hexane for 24 hours. The sample was further washed with hexane for several times. N_2- and air- refers to the atmosphere during thermal treatment.

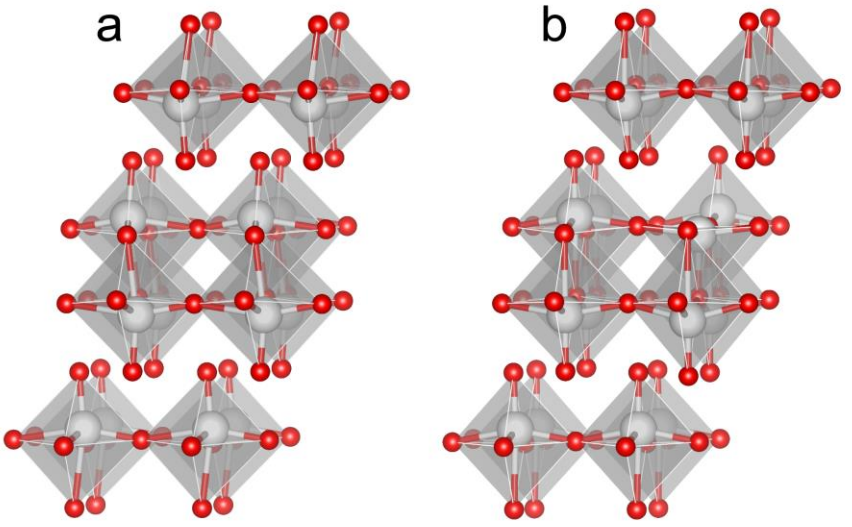

Supplementary Figure 8 Optimized supercells. (a) α-MoO_3 ($Mo_{16}O_{48}$) and (b) α-MoO_{3-x} ($Mo_{16}O_{47}$).

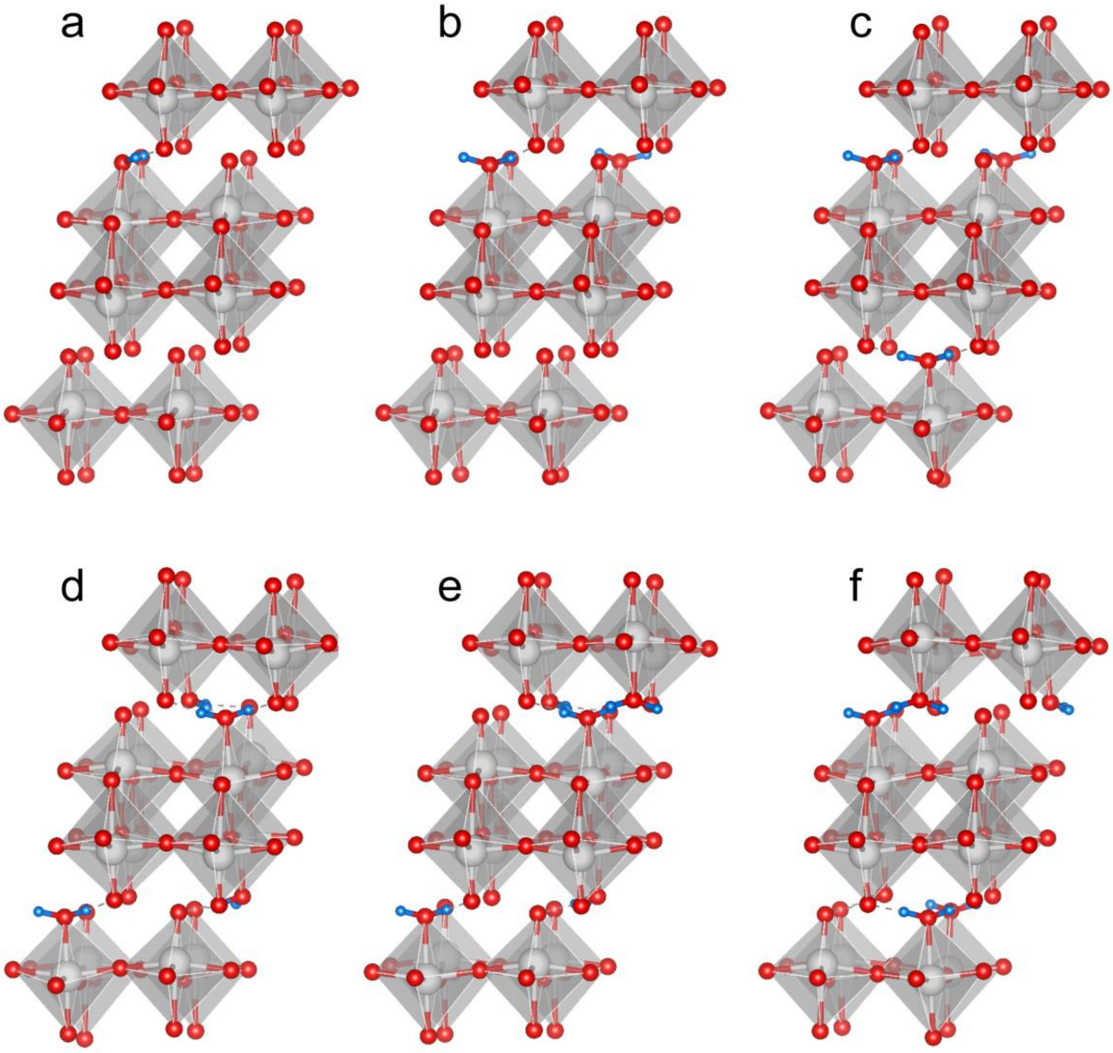

Supplementary Figure 9 Optimized supercells. (a) $Mo_{16}O_{47}\cdot H_2O$, (b) $Mo_{16}O_{46}\cdot 2H_2O$, (c) $Mo_{16}O_{45}\cdot 3H_2O$, (d) $Mo_{16}O_{44}\cdot 4H_2O$, (e) $Mo_{16}O_{43}\cdot 5H_2O$, and (f) $Mo_{16}O_{42}\cdot 6H_2O$.

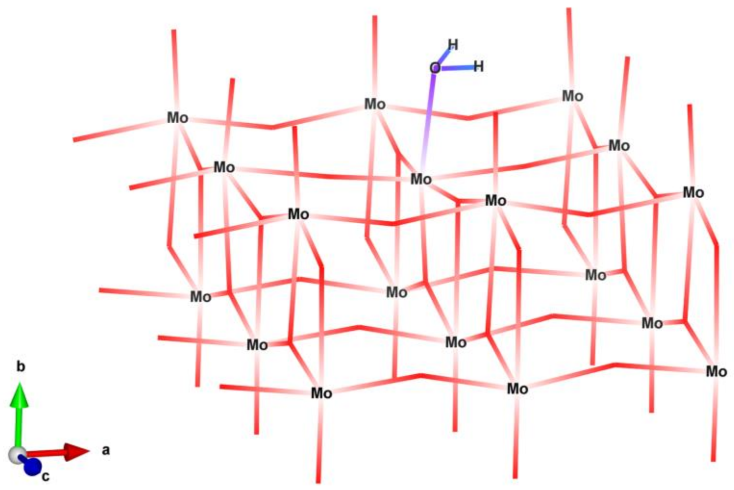

Supplementary Figure 10 The most stable configuration of H_2O-incorporated MoO_3 calculated from DFT.

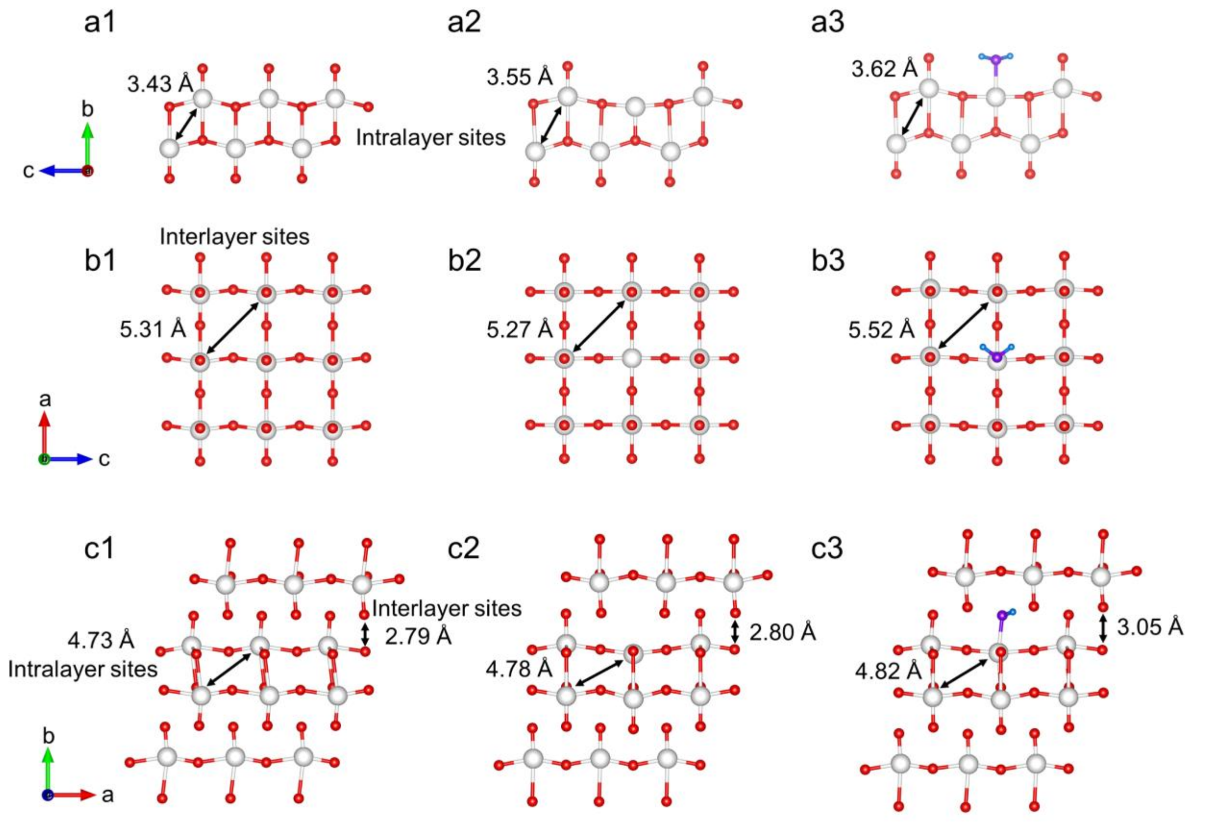

Supplementary Figure 11 Simulated crystal structures. Crystal structures of (a1, b1, c1) MoO_3, (a2, b2, c2) oxygen-deficient MoO_3, and (a3, b3, c3) H_2O-incorperated MoO_3 projected along different crystallographic directions. (a1, a2, a3), (b1, b2, b3) and (c1, c2, c3) are projected along [100], [010], and [001], respectively.

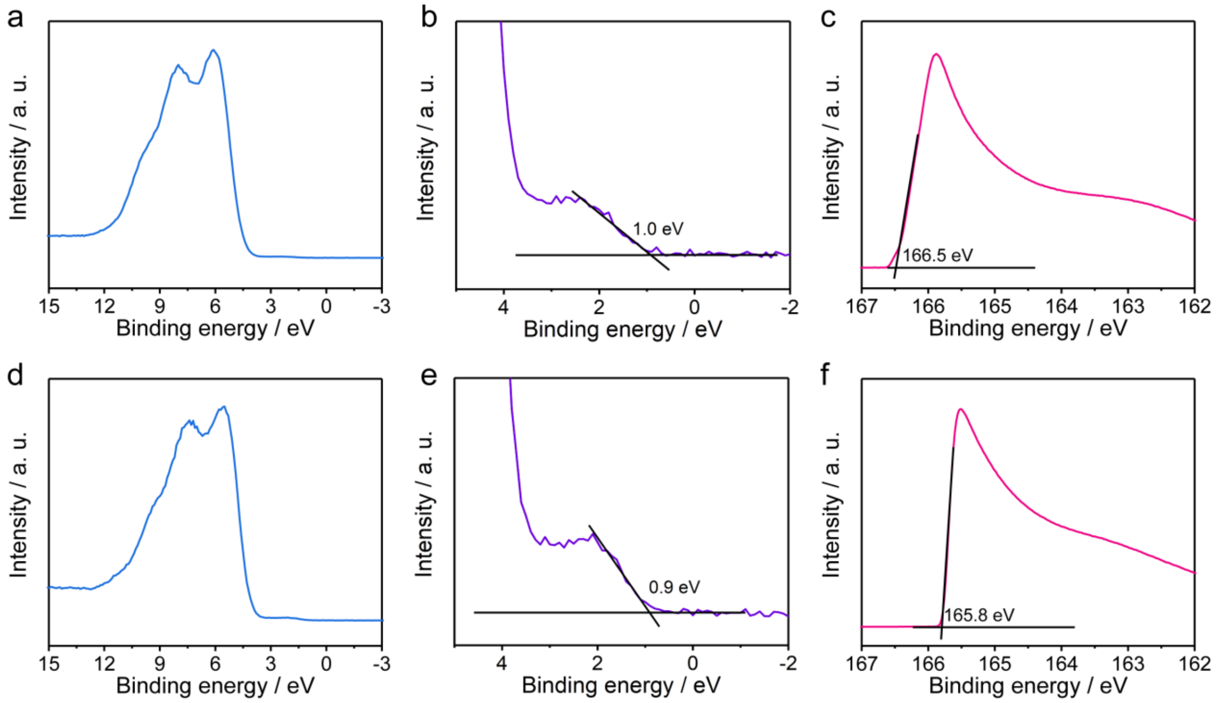

Supplementary Figure 12 UPS results. (a, b) Valance-band spectra and (c) secondary electron cutoff of α-MoO_3. (d, e) Valance-band spectra and (f) secondary electron cutoff of e-MoO_3.

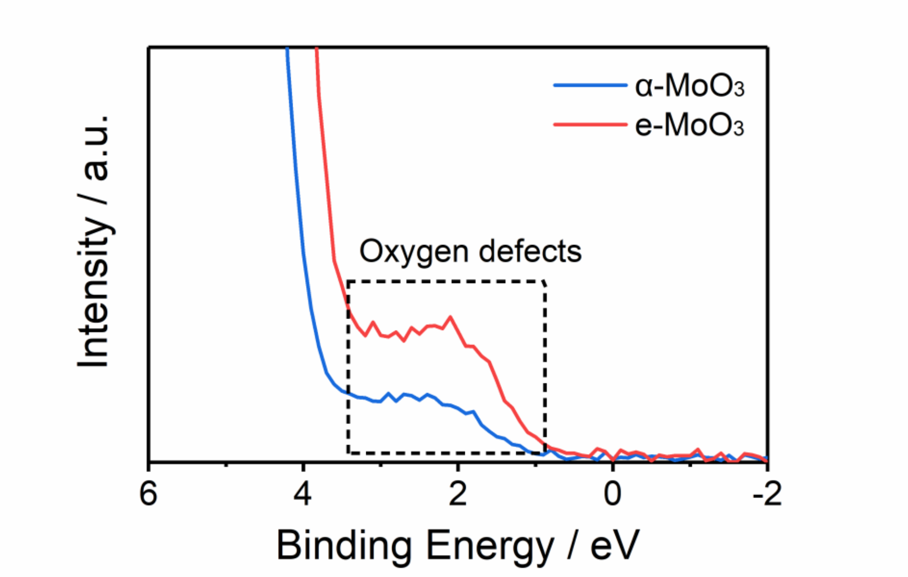

Supplementary Figure 13 Valance band margins of α-MoO_3 and e-MoO_3.

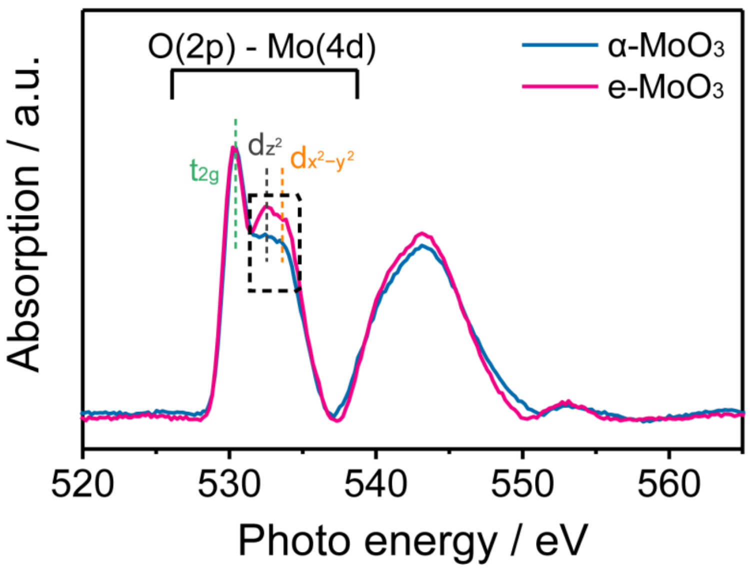

Supplementary Figure 14 NEXAFs O K-edge of α-MoO_3 and e-MoO_3.

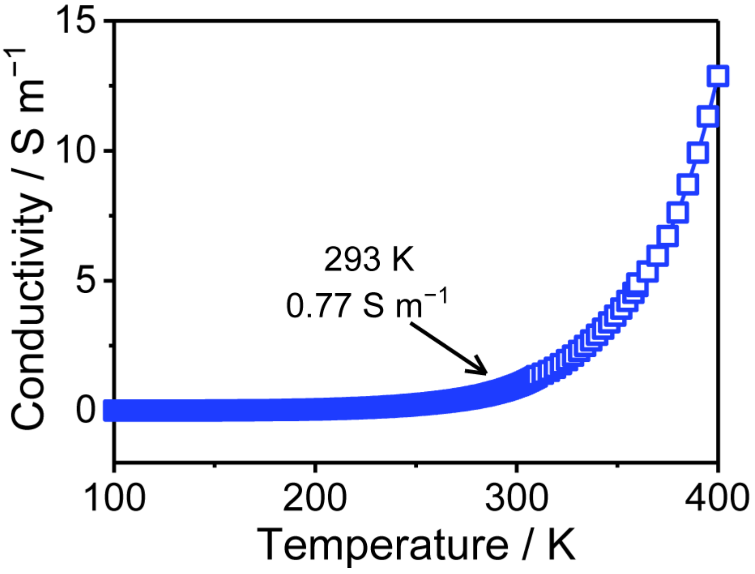

Supplementary Figure 15 Conductivity of e-MoO_3 measured with the Van der Pauw method.

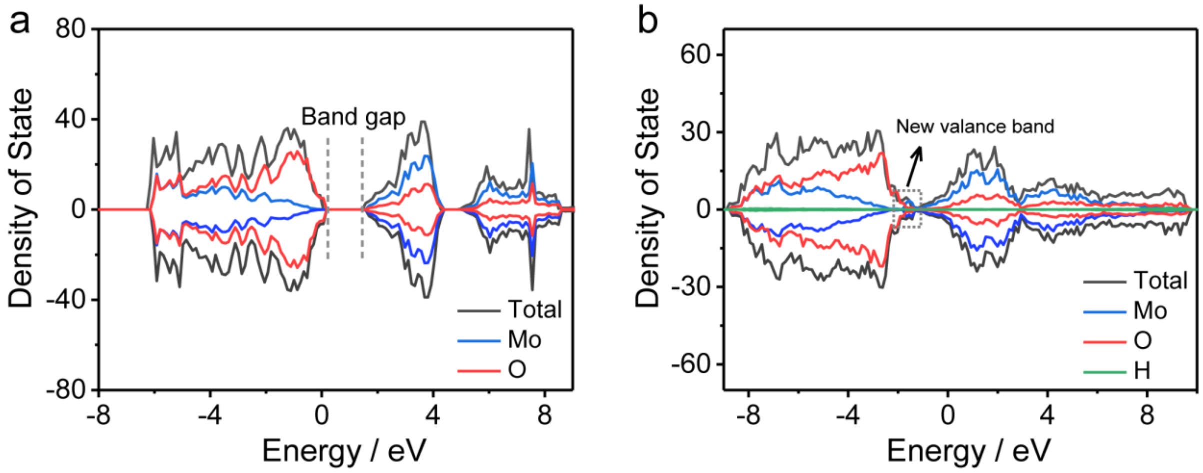

Supplementary Figure 16 Projected density of state. (a) α-MoO_3 and (b) α-$MoO_{3-x}\cdot xH_2O$. Calculation for α-MoO_3 and α-$MoO_{3-x}\cdot xH_2O$ used supercell $Mo_{16}O_{48}$ (Supplementary Fig. 7a) and $Mo_{16}O_{45}\cdot 3H_2O$ (Supplementary Fig. 8c).

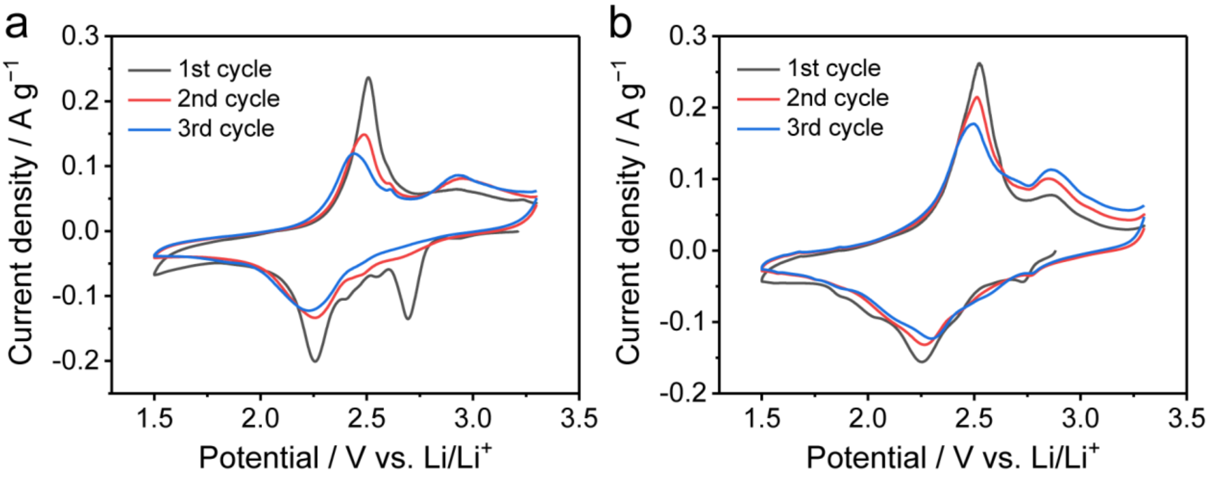

Supplementary Figure 17 The initial three CV cycles at 0.1 mV s^{-1}. (a) α-MoO_3 and (b) e-MoO_3 electrodes.

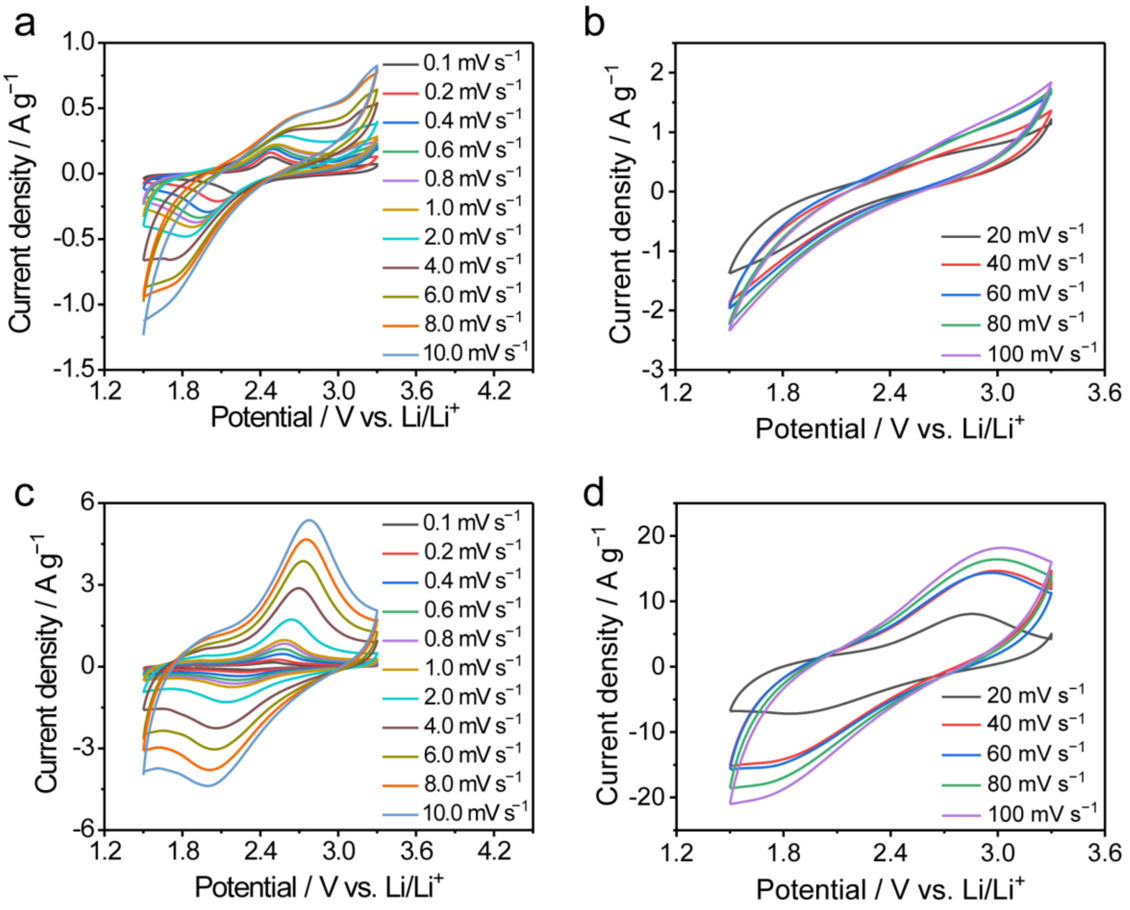

Supplementary Figure 18 CV curves collected at various scan rates. (a, b) α-MoO_3 and (c, d) e-MoO_3 electrodes.

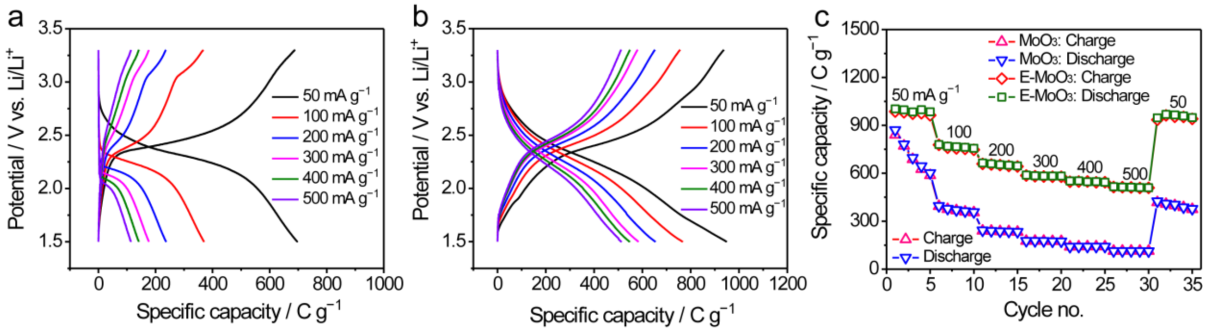

Supplementary Figure 19 Rate tests. Galvanostatic charge/discharge curves at various current densities for (a) α-MoO_3 and (a) e-MoO_3 electrodes. (c) Rate performance of α-MoO_3 and e-MoO_3 electrodes.

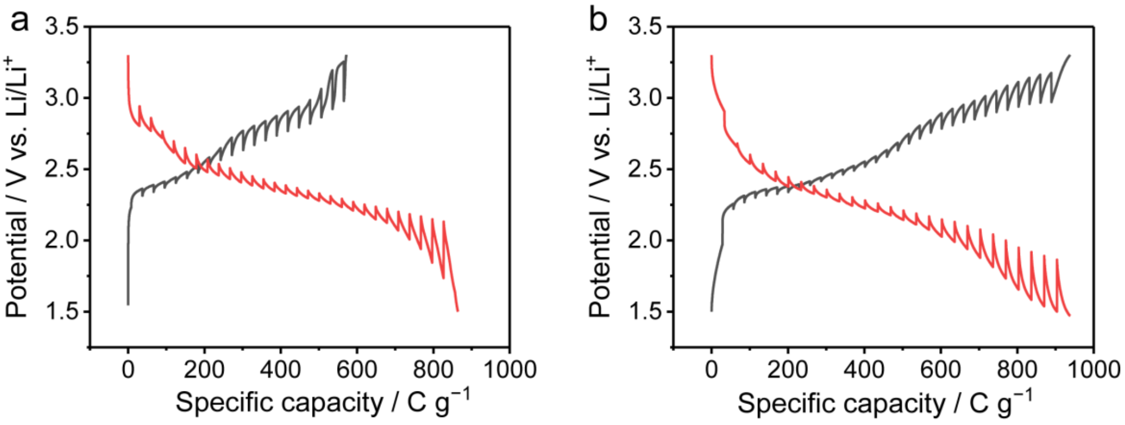

Supplementary Figure 20 GITT charge discharge curves. (a) α-MoO_3 and (b) e-MoO_3 electrodes (50 mA g^{-1} for 10 min, open circuit step for 3 hours).

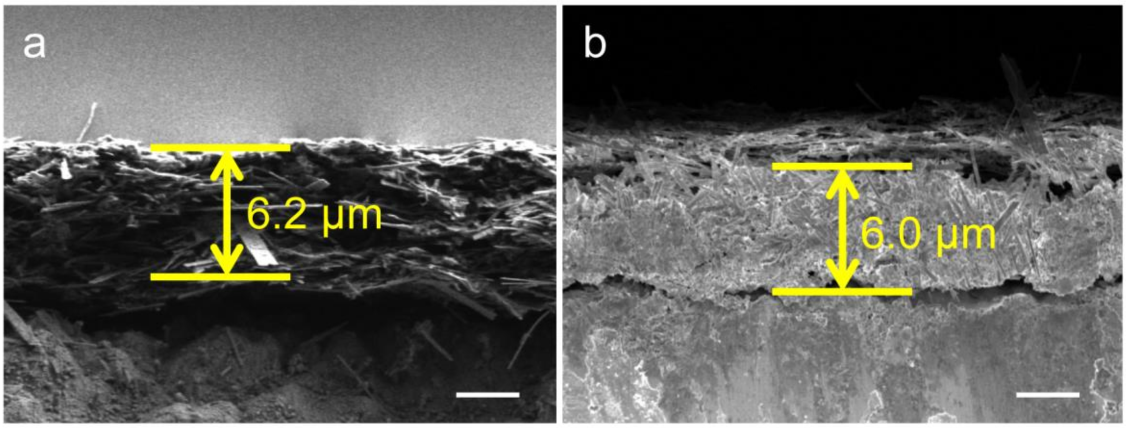

Supplementary Figure 21 Cross sectional SEM images. (a) α-MoO_3 and (b) e-MoO_3 electrodes. The thickness of α-MoO_3 and e-MoO_3 electrodes are determined to be 6.2 and 6.0 μm, respectively. Scale bars, 3 μm.

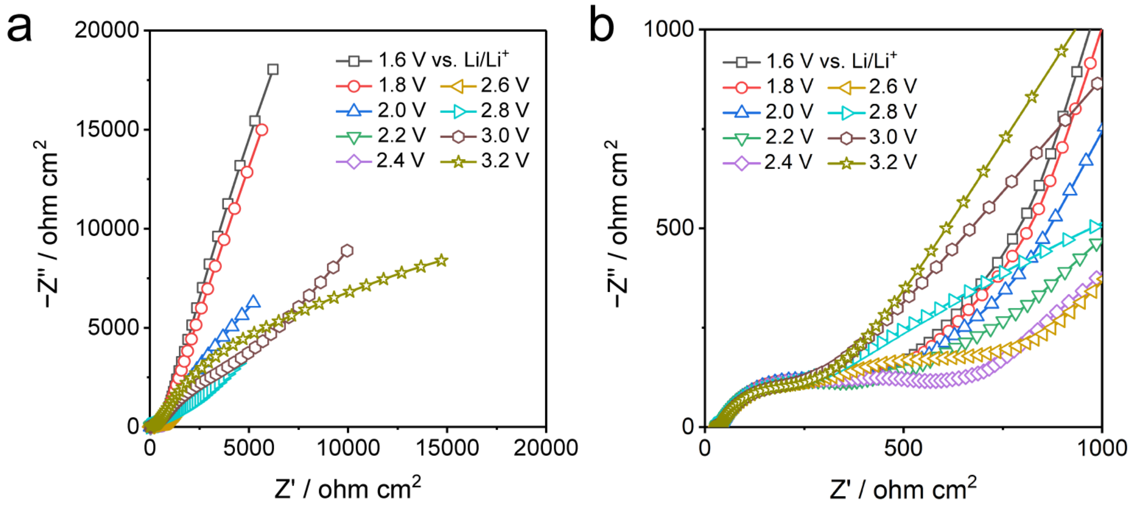

Supplementary Figure 22 Nyquist plots of α-MoO_3 at different potentials.

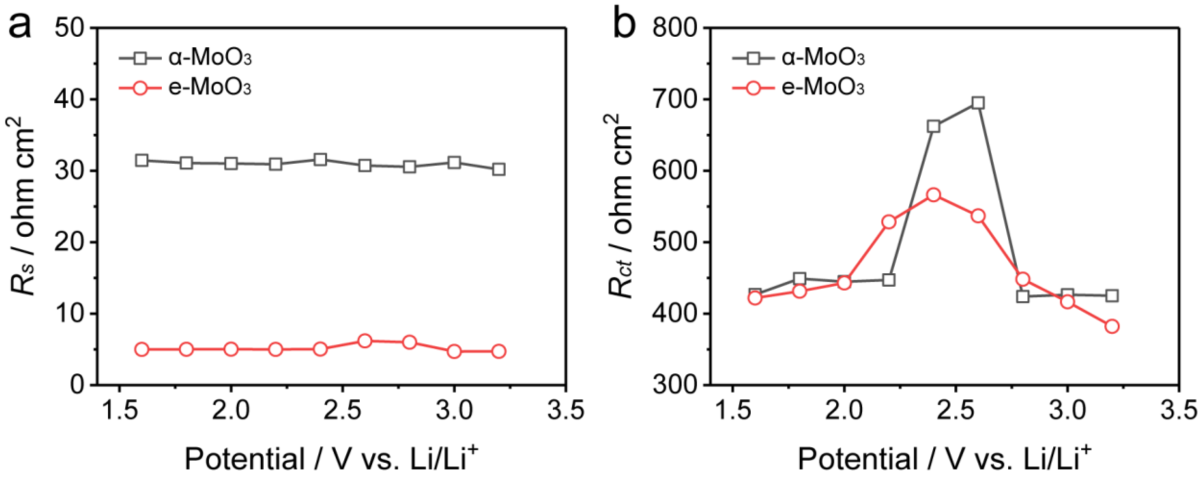

Supplementary Figure 23 EIS results. (a) R_s and (b) R_{ct} of α-MoO_3 and e-MoO_3 electrodes as a function of potential.

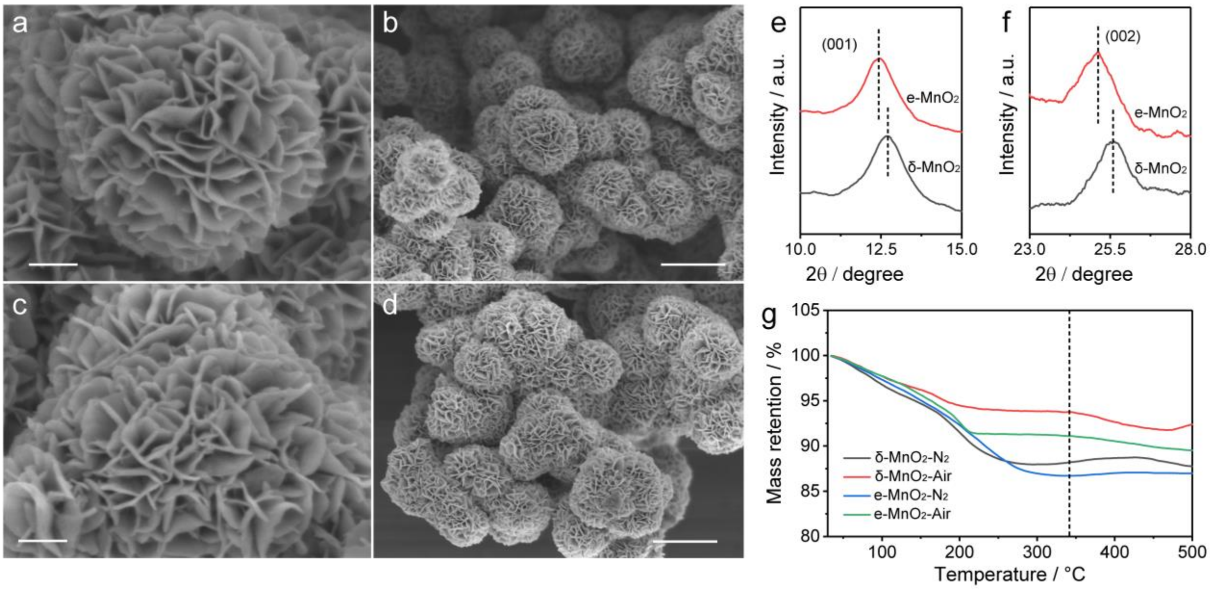

Supplementary Figure 24 Characterizations of δ-MnO_2 and e-MnO_2. SEM images of (a, b) δ-MnO_2 and (c, d) e-MnO_2. Scale bars in (a, c), 100 nm. Scale bars in (b, d), 500 nm. XRD spectra of δ-MnO_2 and e-MnO_2, showing the corresponding e, (001) peak and f, (002) peak. The black line refers to δ-MnO_2, while the red line refers to e-MnO_2. g, TGA curves of δ-MnO_2 and e-MnO_2 measured in nitrogen and air atmosphere.

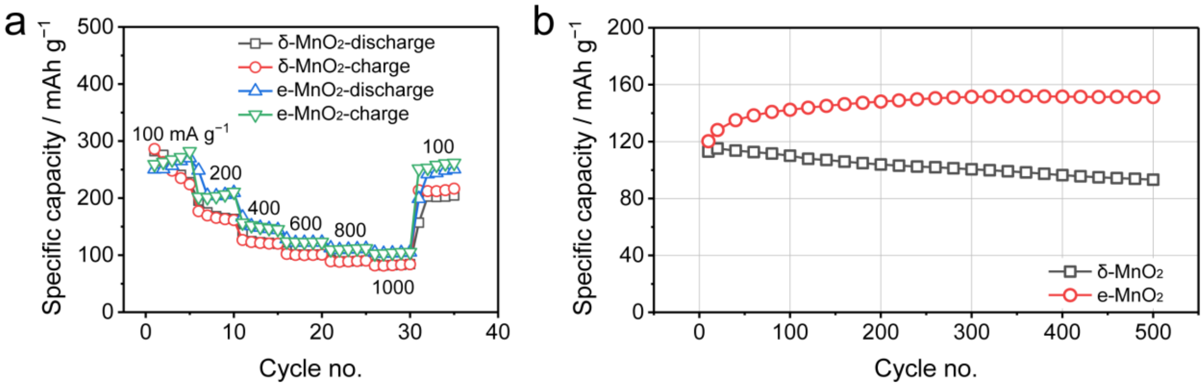

Supplementary Figure 25 Electrochemical performance of δ-MnO_2 and e-MnO_2. (a) Rate performance of δ-MnO_2 and e-MnO_2. (b) Cycling performance of δ-MnO_2 and e-MnO_2 at 1 A g^{-1}.

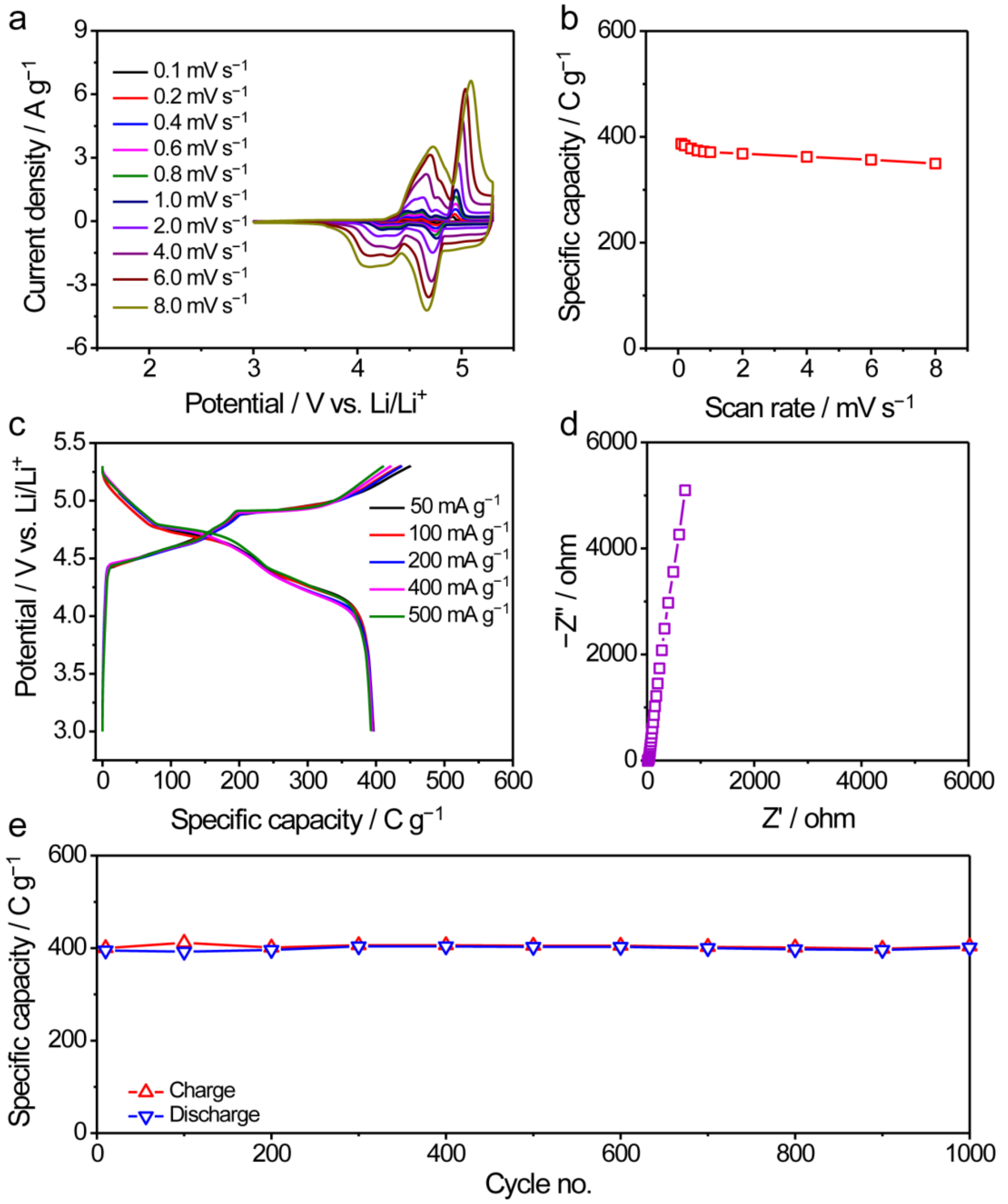

Supplementary Figure 26 Electrochemical performance of the graphite cathode tested in half cell. (a) CV curves collected at various scan rates. (b) Specific capacity as a function of scan rate. (c) Galvanostatic charge/discharge curves measured at different current density. (d) Nyquist plots. (e) Cycling performance at 500 mA g^{-1}.

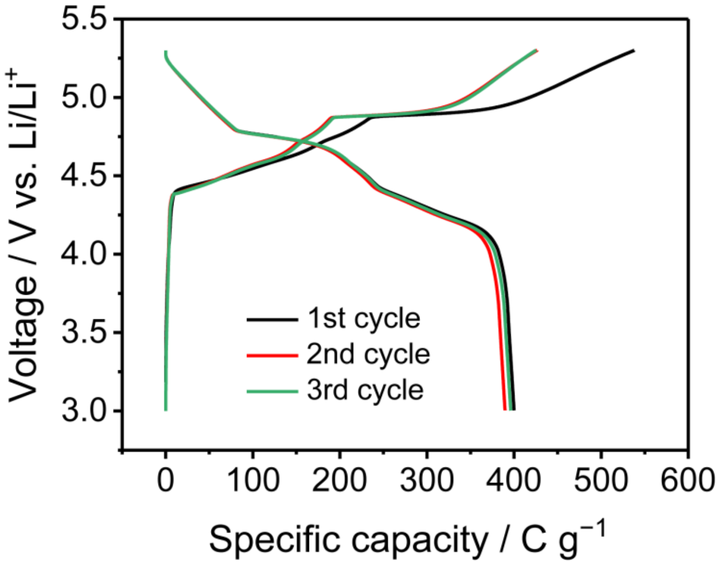

Supplementary Figure 27 The initial three galvanostatic charge/discharge profiles of graphite electrode.

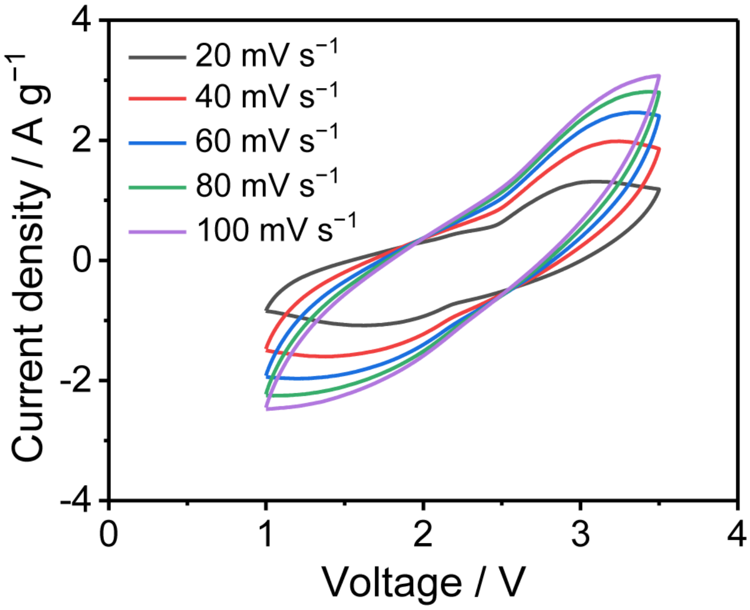

Supplementary Figure 28 CV curves of the DIES device at 20 ~ 100 mV s^{-1}.

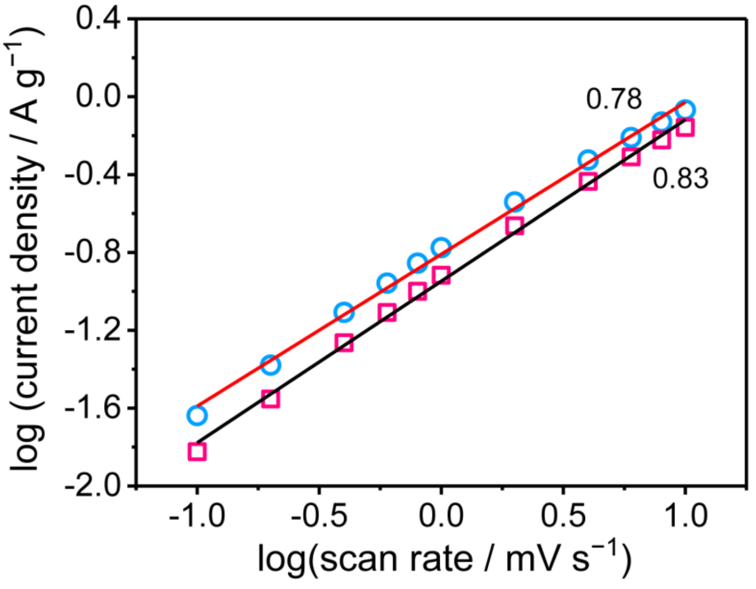

Supplementary Figure 29 The log (current density) versus log(v) plot of the main cathodic and anodic peaks.

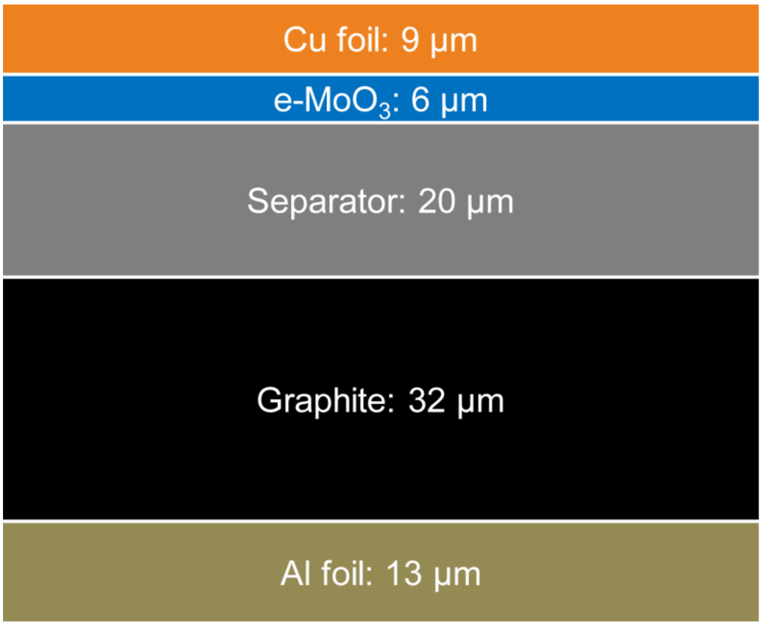

Supplementary Figure 30 Configuration of the full cel1 device.

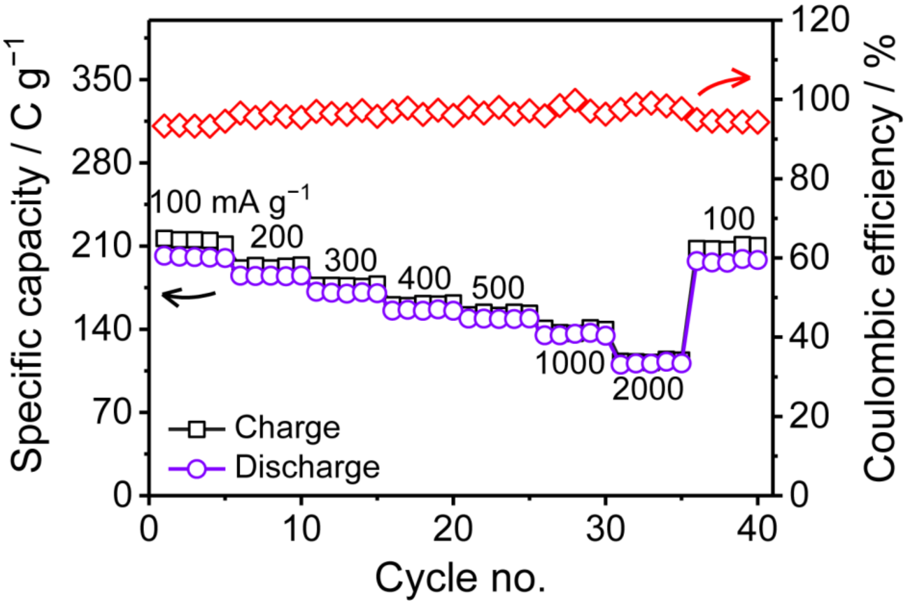

Supplementary Figure 31 Rate performance of e-MoO_3//graphite device based on the mass of active materials.

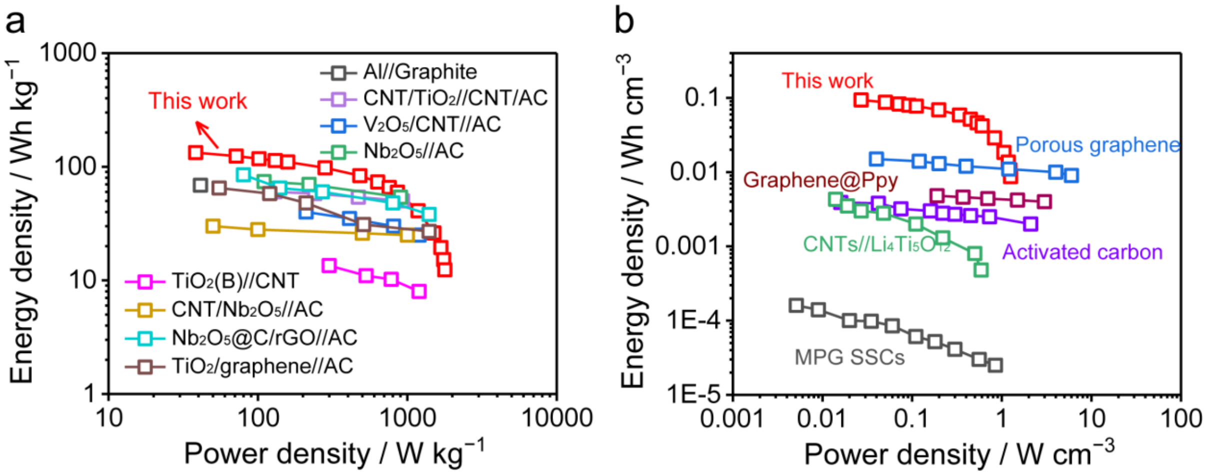

Supplementary Figure 32 Ragon plots. Ragon plots of the e-MoO_3//graphite device based on (a) the mass and (b) the volume of active materials. The gravimetric energy density and power density are compared with state-of-the art energy storage devices like TiO_2(B)//CNT,[1] CNT/TiO_2//CNT/AC,[2] TiO_2/graphene//AC,[3] Nb_2O_5//AC[4], CNT/Nb_2O_5//AC,[5] Nb_2O_5@C/rGO//AC,[6] V_2O_5/CNT//AC,[7] and Al//graphite[8]. The volumetric energy density and power density are compared with literature reported active materials with high volumetric performance, like MPG,[9] graphene@Ppy,[10] CNTs // $Li_4Ti_5O_{12}$,[11] activated carbon,[12] and porous graphene[13].

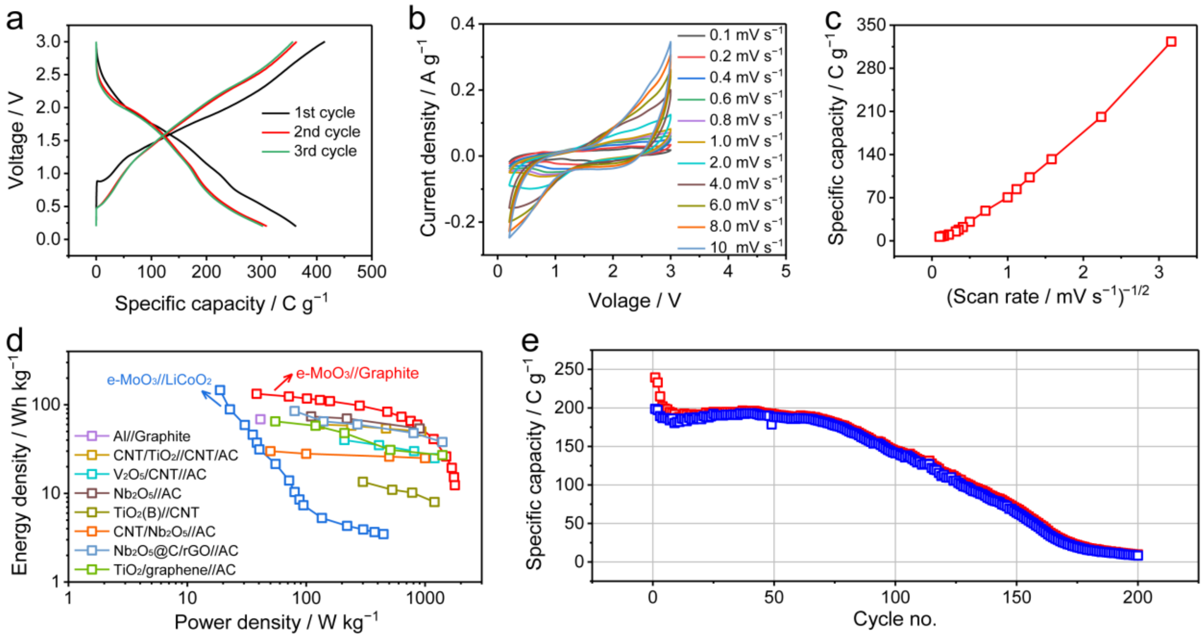

Supplementary Figure 33 Electrochemical performance of the e-MoO_3//$LiCoO_2$ device. (a) The initial three galvanostatic charge/discharge cycles at 100 mA g^{-1}. (b) CV curves of the device at different scan rates. (c) Change of the gravimetric capacity based on the total active mass as a function of $v^{-1/2}$. (d) Ragone plots of the e-MoO_3//$LiCoO_2$ device in comparison with the e-MoO_3//graphite device and state-of-the-art high-rate batteries. (e) Cycling performance of the e-MoO_3//$LiCoO_2$ device at 400 mA g^{-1}.

Supplementary Tables

Supplementary Table 1 Raman peak assignment for MoO_3 and e-MoO_3. The results are also compared with the reported MoO_3 and MoO_{3-x}.

<table>
<tr><th rowspan="2">Type of peaks</th><th colspan="4">Raman shift (cm^{-1})</th></tr>
<tr><th>MoO_3</th><th>e-MoO_3</th><th>Reported MoO_3[14]</th><th>Reported oxygen-deficient MoO_3[15]</th></tr>
<tr><td>Antisymmetric stretching (A_g of Mo=O)</td><td>990</td><td>969</td><td>995</td><td>996</td></tr>
<tr><td>Symmetric stretching (B_{1g} of Mo-O-Mo)</td><td>814</td><td rowspan="2">724</td><td>818</td><td>820</td></tr>
<tr><td>Antisymmetric stretching (B_{2g}, B_{3g} of Mo-O-Mo)</td><td>660</td><td>666</td><td>667</td></tr>
<tr><td>Scissoring modes (B_{1g} of Mo-O-Mo)</td><td>376</td><td rowspan="4">346, 221, 199</td><td>377</td><td>366–380</td></tr>
<tr><td>Bending (A_g, B_{1g} of Mo-O-Mo)</td><td>335</td><td>336</td><td>338</td></tr>
<tr><td>Wagging vibrations (B_{2g}, B_{3g}, Mo=O)</td><td>288</td><td>289</td><td>285</td></tr>
<tr><td>Scissoring mode (B_{3g} of Mo-O-Mo)</td><td>246</td><td>245</td><td>245</td></tr>
<tr><td>Polyhedra along the chain axis (A_g, B_{1g})</td><td>156</td><td>153</td><td>157</td><td>158</td></tr>
<tr><td>Unclear peaks</td><td></td><td>561, 486</td><td></td><td></td></tr>
</table>

Supplementary Table 2 Electrochemical performance of our e-MoO_3 compared with α-MoO_3 and recently reported MoO_3 based electrodes.

Type of electrode	Loading mass	Scan rate or current denstiy	Gravimetric capacity	Areal capacity	Ref
e-MoO_3	0.6 mg cm^{-2}	0.1 mV s^{-1}<br>10 mV s^{-1}	964 C g^{-1}<br>388 C g^{-1}	578 mC cm^{-2}<br>233 mC cm^{-2}	This work
α-MoO_3	0.6 mg cm^{-2}	0.1 mV s^{-1}<br>10 mV s^{-1}	756 C g^{-1}<br>34 C g^{-1}	454 mC cm^{-2}<br>20 mC cm^{-2}	This work
MoO_{3-x}	0.04 mg cm^{-2}	1 mV s^{-1}<br>10 mV s^{-1}	980 C g^{-1}<br>800 C g^{-1}	39 mC cm^{-2}<br>32 mC cm^{-2}	[16]
MoO_3/SWCNTs	0.27 mg cm^{-2}	0.1 mV s^{-1}<br>10 mV s^{-1}	1000 C g^{-1}<br>400 C g^{-1}	270 mC cm^{-2}<br>108 mC cm^{-2}	[15]
MoO_3/functionlized SWCNTs	0.45 mg cm^{-2}	0.1 mV s^{-1}<br>10 mV s^{-1}	698 C g^{-1}<br>334 C g^{-1}	314 mC cm^{-2}<br>150 mC cm^{-2}	[17]
Mesoporous α-MoO_3	low mass loading	1 mV s^{-1}<br>10 mV s^{-1}	600 C g^{-1}<br>500 C g^{-1}	-	[18]
Plasma etched α-MoO_3	Not provided	100 mA g^{-1}	990 C g^{-1}	-	[19]

Supplementary Notes

Supplementary Note 1

In Supplementary Fig. 1, all XRD peaks can be indexed into an orthorhombic α-MoO_3 unit cell with the refined lattice parameters of a = 3.96 (6) Å, b = 13.85 (1) Å, c = 3.70 (5) Å, with space group of Pbnm. The peaks located at 12.7, 23.3, 25.7, 27.3, 29.7, 33.7, 35.5, 39.0, 45.7, 46.3, 49.2, and 64.5° are attributed to (020), (110), (040), (021), (130), (111), (041), (060), (200), (210), (002), and (062) planes.

Supplementary Note 2

As shown in Supplementary Fig. 3a, α-MoO_3 displays three characteristic peaks at 987, 850, and 532 cm^{-1}, corresponding to the stretching vibration of Mo=O, the stretching vibration of Mo-O-Mo, and B_{3u} mode of the stretching vibration of Mo-O-Mo, respectively.[14] By contrast, e-MoO_3 presents two new and apparent peaks associated with the stretching (3412 cm^{-1}) and bending (1622 cm^{-1}) vibration of -OH groups, which originate from the incorporated H_2O in e-MoO_3.[20] Moreover, the peak of e-MoO_3 related to B_{3u} mode of Mo-O-Mo is positively shifted to 539 cm^{-1}, indicating the existence of oxygen vacancies.[21] Meanwhile, the peaks associated with the stretching vibration of Mo=O (968 cm^{-1}) and Mo-O-Mo (827 cm^{-1}) are negatively shifted, which was not observed in the reported oxygen-deficient MoO_3.[21] This result implies the possible coordination of H_2O to Mo atoms, which changes the local structural environment surrounding the Mo atoms. Supplementary Fig. 3b further compares the Raman spectra of α-MoO_3 and e-MoO_3, which reveal that the incorporated H_2O molecules have a significant effect on the structure of e-MoO_3. All the Raman peaks of our α-MoO_3 sample agree well with those of reported α-MoO_3[14,22] (as listed in Supplementary Table 1). Meanwhile, e-MoO_3 shows a remarkable change in its Raman peaks compared with MoO_3[14] and the previously reported oxygen-deficient MoO_3[15]. This result further supports that the incorporation of H_2O disturbs the crystal arrangement of the [MoO_6] octahedra bilayers.

Supplementary Note 3

As observed in Supplementary Fig. 7, the rectangle frame indicates the peaks corresponding to Li compounds in Li-MoO_3, Air-LiMoO_3, and N_2-LiMoO_3. However, these peaks are not found in e-MoO_3 based samples, which implies Li was removed from the crystal lattice during sonication and wash in water.

Supplementary Note 4

Supplementary Fig. 10 illustrates the most stable configuration, in which H_2O occupies the sites corresponding to oxygen anions connected to Mo cation. The incorporated H_2O induces the distortion of the [MoO_6] octahedra, leading to substantial changes in typical O-O distances (both intra and inter-layer ones). Supplementary Fig. 11 compares the structures of MoO_3, oxygen-deficient MoO_3, and H_2O-incorporated MoO_3 projected along different crystallographic directions, in which the calculated O-O distances are displayed. It is revealed that H_2O-incorporated MoO_3 presents the largest spaces for Li-ion storage/diffusion sites.[23] In combination with the in-operando XRD analysis, the improved rate capability and cycling stability of e-MoO_3 can be attributed to (1) the enlarged spaces for Li-ion storage/diffusion sites, which accelerate the Li^+ diffusion and prevent irreversible phase transition of e-MoO_3 during the initial lithiation, and (2) the shielding effect of the incorporated H_2O, which diminishes the electrostatic interaction between Li^+ and MoO_3 lattice[24,25], allows a lower ion-diffusion energy barrier, and alleviates the volume change of e-MoO_3 during charge and discharge.

Supplementary Note 5

An excitation of 168.9 eV was utilized in the measurements of valence band structure and secondary electron cutoff. The binding energy (BE) was calibrated and referenced to the E_f of Au foil. The work function (Φ) of the sample was determined according to the equation $\Phi = h\nu - \Delta E$, where ΔE is the spectral width, i.e., the energy difference between the secondary electron cutoff and the sample Fermi level. In order to obtain the secondary electron cutoff, a −10 V bias was applied to the sample, which accelerated all the photoelectrons with higher kinetic energy to overcome the work function of the analyzer.

As shown in Supplementary Fig. 12, the valence band margin of α-MoO_3 is 1.0 eV below the Fermi level, and the second electron cutoff is located at 166.5 eV. Thus, the work function is determined to be 3.4 eV for α-MoO_3. By contrast, the work function of e-MoO_3 is calculated to be 4.0 eV, which is slightly higher than that of α-MoO_3. Moreover, the increment in band density near the Fermi level was reported to be assigned to the oxygen defects (Supplementary Fig. 13).[26]

Supplementary Note 6

Supplementary Fig. 14 compares the O K-edge NEXAFs spectra of α-MoO_3 and e-MoO_3. The high-intensity peak within 528–537 eV originates from the hybridization of O 2p with Mo 4d states, which is split into the

t_{2g} and e_g (dz^2, $dx^2{-}y^2$) like peaks. In comparison with the e_g peak of α-MoO_3, the intensity increment in the e_g peak of e-MoO_3 is because of the increased density of conduction band by oxygen vacancies.[27,28]

Supplementary Note 7

Compared with α-MoO_3, α-$MoO_{3-x}\cdot xH_2O$ shows an additional valance band near valance band maximum, which shows a typical Mo-d characteristic (Supplementary Fig. 16). This newly formed band bridges the gap between valance band and conduction band, thus explains for the greatly improved conductivity of our e-MoO_3 sample. Of note is that this newly formed valance band is in good agreement with the increment in valance band observed in Supplementary Fig. 13.

Supplementary Note 8

δ-MnO_2 nanoflowers were prepared through a solution based refluxing method. Briefly, 10 mmol of $MnSO_4\cdot H_2O$ was dissolved in 300 mL distilled water, followed by adding 25 mmol of $KMnO_4$. The obtained homogeneous solution was refluxed under continuous magnetic stirring at 150 °C for 6 hours. Upon cooling to room temperature, the product was washed with distilled water for three times and dried under vacuum at 80 °C. e-MnO_2 was obtained by dispersing 500 mg of δ-MnO_2 in 16 mL of 0.8 M n-butyl lithium in hexane. The mixture was then stirred for 24 hours at room temperature under the protection of inert argon atmosphere. The lithiated compound was separated by vacuum filtration and washed several times with hexane. Subsequently, the sample was placed in 100 mL of distilled water and stirred for half an hour. Finally, the sample was centrifuged and dried in a vacuum oven at 80 °C.

SEM images show almost no difference between the morphologies of initial δ-MnO_2 (Supplementary Fig. 24a and b) and treated δ-MnO_2 (denoted as e-MnO_2, Supplementary Fig. 24c and d). The slight vdW gap expansion of e-MnO_2 was evidenced by the XRD peak shift observed for e-MnO_2 compared with δ-MnO_2 (Supplementary Fig. 24e and f). In detail, the (001) and (002) peaks of e-MnO_2 are negatively shifted by 0.3° and 0.6°, respectively. TGA (Supplementary Fig. 24g) of δ-MnO_2 and e-MnO_2 in air was conducted to estimate the amount of incorporated H_2O in the whole sample. Both samples present the loss of intercalated water and the re-oxidation of oxygen-deficient MnO_2 below 350 °C,[29] which are indicative of $MnO_{1.84}\cdot 0.58\ H_2O$ and $MnO_{1.82}\cdot 0.70\ H_2O$ for δ-MnO_2 and e-MnO_2, respectively. Admittedly, the effect of our strategy on δ-MnO_2 is not significant as it is on α-MoO_3, in terms of the vdW gap expansion degree and the amount of incorporated H_2O. However, we believe that e-MnO_2 can be further optimized by adjusting parameters related

to material post-treatment (temperature, concentration of n-butyllithium, treatment time, and so on).

The electrochemical performance of δ-MnO_2 and e-MnO_2 as cathodes (loading mass of 1.6 mg cm^{-2}) for Zn-ion aqueous batteries was assessed in a two-electrode system with Zn foil as anode (Supplementary Fig. 25). To prepare the electrodes, active materials (δ-MnO_2 and e-MnO_2), binder (polyvinylidene fluoride) and Super P carbon black were mixed with a weight ratio of 8:1:1. The mixture was dispersed in N-methyl pyrrolidone and coated on a piece of carbon cloth, followed by drying at 80 °C for 12 hours under vacuum. As shown in Supplementary Fig. 25a, the specific capacity of δ-MnO_2 decays fast from 286 to 224 mAh g^{-1} during the first five charge/discharge cycles at 100 mA g^{-1}. In contrast, e-MnO_2 depicts gradually increasing capacity from 250 to 270 mAh g^{-1} during the first five cycles at 100 mA g^{-1}. When the current density is increased to 1 A g^{-1}, the specific capacities of δ-MnO_2 and e-MnO_2 are 82 and 102 mAh g^{-1}, indicating the better rate capability of e-MnO_2 than that of δ-MnO_2. Moreover, e-MnO_2 shows better cycling performance than δ-MnO_2 (Supplementary Fig. 25b). After 500 cycles at 1 A g^{-1}, the capacity of e-MnO_2 first increases from 120 to 150 mAh g^{-1} and then keeps almost stable, while the capacity of δ-MnO_2 gradually decreases from 113 to 93 mAh g^{-1}. All these results imply the feasibility of our H_2O-incorporation strategy in improving the charge storage ability of birnessite δ-MnO_2.

Supplementary Note 9

Identified by both CV and galvanostatic charge/discharge curves (Supplementary Fig. 26), the anion intercalation into graphite displays high kinetic with a potential window of 3.5-5.3 V *vs.* Li^+/Li. The capacity reaches 387 C g^{-1} at 0.1 mV s^{-1}, while 350 C g^{-1} is retained at 8 mV s^{-1}. Moreover, the capacity almost keeps the same during galvanostatic charge/discharge tests when the current density increased from 50 mA g^{-1} (capacity of 396 C g^{-1}) to 500 mA g^{-1} (capacity of 392 C g^{-1}). Graphite also demonstrates outstanding cycling stability with almost no capacity decay after 1000 charge/discharge cycles at 500 mA g^{-1}.

Supplementary Note 10

Supplementary Fig. 29 presents the plots of log (i) versus log (v) from 0.1 to 10 mV s^{-1} for the main redox peaks. Following equation (1), the charge storage kinetics can be also reflected by determining b-value. It is well established that a b-value of 0.5 is indicative of a diffusion-controlled process, while a b-value of 1.0 identifies a surface-controlled process. As revealed, the b-values for anodic and cathodic peaks are determined as 0.83 and 0.78, which further confirms the combination of diffusion-related contribution and

capacitive contribution.

$$i = av^{b} \qquad (1)$$

Supplementary Note 11

As shown in Supplementary Fig. 30, mass loading for e-MoO_3 is 0.6 mg cm^{-2}, while the mass loading for graphite is 1.5 mg cm^{-2}. Since the loading mass of graphite is 2.5 times of that of e-MoO_3, as well as the volume of graphite, is more than 5 times of that of e-MoO_3 electrode, we conclude it would be further beneficiary to the full-cell energy density and power density if the cathode with comparable capacity could be developed.

Supplementary Note 12

The full e-MoO_3//$LiCoO_2$ Li-ion cell was assembled by using a two-electrode stainless steel Swagelok cell. e-MoO_3 coated on Cu foil and $LiCoO_2$ coated on Al foil were used as anode and cathode, respectively, with a mass ratio of 1:2. Supplementary Fig. 33a presents the first three charge/discharge cycles of the e-MoO_3//$LiCoO_2$ device at 100 mA g^{-1}, revealing a reversible specific capacity of 309 C g^{-1} for the e-MoO_3//$LiCoO_2$ device within a voltage window of 0.2-3 V. CV curves were collected for the e-MoO_3//$LiCoO_2$ device at scan rates from 0.1 to 100 mV s^{-1} (Supplementary Fig. 33b). Supplementary Fig. 33c plots the specific capacity as a function of $v^{-1/2}$, which shows a close-to-linear shape. This means that the specific capacity of the device is significantly limited by the solid-state diffusion, and is dependent on the scan rate. This behavior is apparently different from the e-MoO_3//graphite device, whose capacity is mostly independent on the scan rate. The Ragone plots of e-MoO_3//$LiCoO_2$ are calculated and compared with those of e-MoO_3//graphite and other reported cation-ion batteries (Supplementary Fig. 33d). Although the maximum energy density of e-MoO_3//$LiCoO_2$ device (146 Wh kg^{-1}) is slightly higher than that of e-MoO_3//graphite device (133 Wh kg^{-1}), the power density of e-MoO_3//$LiCoO_2$ is far below that of e-MoO_3//graphite device. Finally, the cycling stability of the e-MoO_3//$LiCoO_2$ device was assessed at 400 mA g^{-1} (Supplementary Fig. 33e). The device can be stably operated for about only 60 cycles. Afterwards, the capacity decays very fast at a capacity decay rate of 1.28 C g^{-1} $cycle^{-1}$. On the other hand, by employing commercial $LiNi_{1-x-y}Co_xMn_yO_2$ (NCM) as cathode, the fabricated full Li-ion cell based on e-MoO_3 is hoped to achieve slightly higher energy density and better cycling stability. However, the power density is predicted to be still much lower than our e-MoO_3//graphite device, as the rate performance of NCM is significantly

lower than anion-intercalation graphite. Therefore, we conclude that the limitation of constructing high-rate Li-ion batteries based on e-MoO_3 anode lies in the unsatisfying performance of cathode, especially the rate performance. Developing cation-intercalation cathode with high discharge potential, large rate capability and long-term cycling stability would be beneficial for further boosting the performance of Li-ion batteries based on e-MoO_3 anode.

Supplementary References

1. Wang, Q., Wen, Z. H. & Li, J. H. A Hybrid Supercapacitor Fabricated with a Carbon Nanotube Cathode and a TiO_2–B Nanowire Anode. *Adv. Funct. Mater.* **16**, 2141–2146 (2006).

2. Chen, Z. *et al.* 3D nanocomposite architectures from carbon-nanotube-threaded nanocrystals for high-performance electrochemical energy storage. *Adv. Mater.* **26**, 339–345 (2014).

3. Le, Z. *et al.* Pseudocapacitive Sodium Storage in Mesoporous Single-Crystal-like TiO_2-Graphene Nanocomposite Enables High-Performance Sodium-Ion Capacitors. *ACS Nano* **11**, 2952–2960 (2017).

4. Lim, E. *et al.* Advanced hybrid supercapacitor based on a mesoporous niobium pentoxide/carbon as high-performance anode. *ACS Nano* **8**, 8968–8978 (2014).

5. Wang, X. L. *et al.* High-Performance Supercapacitors Based on Nanocomposites of Nb_2O_5 Nanocrystals and Carbon Nanotubes. *Adv. Energy Mater.* **1**, 1089–1093 (2011).

6. Lim, E. *et al.* High-Performance Sodium-Ion Hybrid Supercapacitor Based on Nb_2O_5@Carbon Core-Shell Nanoparticles and Reduced Graphene Oxide Nanocomposites. *Adv. Funct. Mater.* **26**, 3711–3719 (2016).

7. Chen, Z. *et al.* High-performance supercapacitors based on intertwined CNT/V_2O_5 nanowire nanocomposites. *Adv. Mater.* **23**, 791–795 (2011).

8. Wang, D. Y. *et al.* Advanced rechargeable aluminium ion battery with a high-quality natural graphite cathode. *Nat. Commun.* **8**, 14283 (2017).

9. Wu, Z. S., Parvez, K., Feng, X. & Mullen, K. Graphene-based in-plane micro-supercapacitors with high power and energy densities. *Nat. Commun.* **4**, 2487 (2013).

10. Fan, Z. *et al.* High Density of Free-Standing Holey Graphene/PPy Films for Superior Volumetric Capacitance of Supercapacitors. *ACS Appl. Mater. Interfaces* **9**, 21763–21772 (2017).

11. Zuo, W., Wang, C., Li, Y. & Liu, J. Directly grown nanostructured electrodes for high volumetric energy density binder-free hybrid supercapacitors: a case study of CNTs//$Li_4Ti_5O_{12}$. *Sci. Rep.* **5**, 7780 (2015).

12. Acerce, M., Voiry, D. & Chhowalla, M. Metallic 1T phase MoS_2 nanosheets as supercapacitor electrode materials. *Nat. Nanotechnol.* **10**, 313–318 (2015).

13. Tao, Y. *et al.* Towards ultrahigh volumetric capacitance: graphene derived highly dense but porous carbons for supercapacitors. *Sci. Rep.* **3**, 2975 (2013).

14. Liang, R., Cao, H. & Qian, D. MoO_3 nanowires as electrochemical pseudocapacitor materials. *Chem. Commun.* **47**, 10305–10307 (2011).

15. Mendoza-Sánchez, B. *et al.* An investigation of the energy storage properties of a 2D α-MoO_3-SWCNTs composite films. *2D Mater.* **4**, 015005 (2016).

16. Kim, H. S. *et al.* Oxygen vacancies enhance pseudocapacitive charge storage properties of MoO_{3-x}. *Nat. Mater.* **16**, 454–460 (2017).

17. Mendoza-Sánchez, B. & Grant, P. S. Charge storage properties of a α-MoO_3/carboxyl-functionalized single-walled carbon nanotube composite electrode in a Li ion electrolyte. *Electrochim. Acta* **98**, 294–302 (2013).

18. Brezesinski, T., Wang, J., Tolbert, S. H. & Dunn, B. Ordered mesoporous alpha-MoO_3 with iso-oriented nanocrystalline walls for thin-film pseudocapacitors. *Nat. Mater.* **9**, 146–151 (2010).

19. Zhang, G. *et al.* α-MoO_{3-x} by plasma etching with improved capacity and stabilized structure for lithium storage. *Nano Energy* **49**, 555–563 (2018).

20. Wang, X.-J., Nesper, R., Villevieille, C. & Novák, P. Ammonolyzed MoO_3 Nanobelts as Novel Cathode Material of Rechargeable Li-Ion Batteries. *Adv. Energy Mater.* **3**, 606–614 (2013).

21. Sun, Z. *et al.* Largely enhanced electrochemical performance in MoO_{3-x} nanobelts formed by a "sauna reaction": Importance of oxygen vacancies. *Electrochim. Acta* **239**, 16–24 (2017).

22. Dieterle, M., Weinberg, G. & Mestl, G. Raman spectroscopy of molybdenum oxides. *Phys. Chem. Chem. Phys.* **4**, 812–821 (2002).

23. T. Tsumura, M. I. Lithium insertion/extraction reaction on crystalline MoO_3. *Solid State Ionics* **104**, 183–189 (1997).

24. Lee, H. J., Shin, J. & Choi, J. W. Intercalated Water and Organic Molecules for Electrode Materials of Rechargeable Batteries. *Adv. Mater.* **30**, e1705851 (2018).

25. Kundu, D., Adams, B. D., Duffort, V., Vajargah, S. H. & Nazar, L. F. A high-capacity and long-life aqueous rechargeable zinc battery using a metal oxide intercalation cathode. *Nat. Energy* **1**, 16119 (2016).

26. Nakayama, Y. *et al.* Origins of Improved Hole-injection Efficiency by the Deposition of MoO_3 on the Polymeric Semiconductor Poly (dioctylfluorene-alt-benzothiadiazole). *Adv. Funct. Mater.* **19**, 3746–3752 (2009).

27. Jeong, S. H., Park, B. N., Lee, S. B. & Boo, J. H. Metal-doped ZnO thin films: Synthesis and characterizations. *Sur. Coat. Tech.* **201**, 5318–5322 (2007).

28. Braun, A. *et al.* Nitrogen Doping of TiO_2 Photocatalyst Forms a Second eg State in the Oxygen 1s NEXAFS Pre-edge. *J. Phys. Chem. C* **114**, 516–519 (2009).

29. Jeon, K. M., Cho, J. S. & Kang, Y. C. Electrochemical properties of MnS–C and MnO–C composite powders prepared via spray drying process. *J. Power Sources* **295**, 9–15 (2015).
