# Supplementary material for: Interlayer gap widened α-phase molybdenum trioxide as high-rate anodes for dual-ion-intercalation energy storage devices
Source: Nat Commun. 2020 Mar 12;11:1348. doi: 10.1038/s41467-020-15216-w (PMC7067814; doi:10.1038/s41467-020-15216-w)
Supplement: Supplementary file 2 — Peer Review File [file 41467_2020_15216_MOESM2_ESM.pdf]

Editorial Note: This manuscript has been previously reviewed at another journal that is not operating a transparent peer review scheme. This document only contains reviewer comments and rebuttal letters for versions considered at Nature Communications .

REVIEWERS' COMMENTS:

Reviewer #1 (Remarks to the Author):

The authors have addressed the comments made from a previous review. The paper can be accepted.
